# Review on role of honey in disease prevention and treatment through modulation of biological activities

**DOI:** 10.1515/biol-2025-1069

**Published:** 2025-03-07

**Authors:** Arshad Husain Rahmani, Ali Yousif Babiker

**Affiliations:** Department of Medical Laboratories, College of Applied Medical Sciences, Qassim University, Buraydah 51452, Saudi Arabia

**Keywords:** honey, inflammation, oxidative stress, apoptosis, wound healing, cancer, pathogenesis

## Abstract

Honey has been used for centuries due to its health-promoting properties. Honey and its bioactive compounds regulate oxidative stress, inflammation, and other biological activities, making it a promising natural remedy. Its role as anti-diabetic, wound healing, cardioprotective, anti-microbial, and hepatoprotective potential has been proven through *in vitro* and *in vivo* studies. Moreover, its role in disease management has been reported through the inhibition of pro-inflammatory enzymes and downregulation of pro-inflammatory cytokine expression and secretion. Besides, it exerts modulatory actions on various signaling pathways such as nuclear factor-κB, tumor suppressor genes, apoptosis, angiogenesis, and MAPK pathway. The main aim of this review is to present a wide-ranging overview of the current evidence regarding the impact of honey on the management of various pathogenic conditions. However, further research based on animal models and clinical trials is required to fully understand the mechanisms of action and safety in the management of various diseases. There is limited clinical data on honey and its mechanisms of action. However, comprehensive clinical studies are needed to fully investigate the potential health benefits of honey, including its efficacies, safety, bioavailability, and underlying mechanisms of action.

## 1 Introduction

Natural products have renowned importance in disease treatment because of their versatile approach to health management without any adverse complications. These products also show an imperative approach to cure diseases via the regulation of molecular pathways that are directly or indirectly involved in disease development. It is worth noting that natural products contain a variety of beneficial constituents, including polyphenols, flavonoids, minerals, and vitamins. These constituents have been found to play a crucial role in preventing pathogenesis by modulating several biological activities. Previous studies have confirmed the disease-preventive ability of various natural products by modulating numerous biological activities [1,2,3,4,5].

The seed of the flax plant, an annual herb belonging to the Linaceae family, is known as flaxseed. Consuming flaxseed has been associated with a number of possible health advantages [6]. Some metabolic syndrome (MetS)-related metrics improved more after 12 weeks of flaxseed administration [7]. Supplementing with probiotics, and prebiotics, appears to be a potential strategy for intervention of oxidative stress and cardiometabolic parameters in chronic kidney disease patients [8]. Therapeutic effects of natural products have been further confirmed through several studies [9].

In this vista, honey plays a very effective role in health maintenance, and its disease cure benefit has been practiced in traditional medicine. Honey possesses multifaceted clinical applications in the medical field. Its diverse therapeutic properties and minimal adverse effects make it an attractive option for various conditions. Honey shows apoptotic effects in colon cancer cells [10] and has antineoplastic activity [11]. Further, honey may be useful and has protective effects for the treatment of various disease conditions such as diabetes mellitus (DM), respiratory, gastrointestinal, cardiovascular, and nervous systems, due to the presence of antioxidant in honey [12].

There are numerous clinical uses for honey in the medical industry. It is a desirable alternative for a number of ailments due to its varied therapeutic qualities and low side effects. However, more thorough clinical trials, creating standardized protocols, and taking regional and economic variables into account are advised [13]. In addition, the sustainability of the environment, food security, and safety are all at risk when honey is consumed that has been tampered with or tainted. Further, contaminated honey can cause genetic defects, allergic reactions, and cancerous effects. Regulating the minimum permissible quantities of pollutants in honey is unfortunately illegal at the moment [14]. It is recommended to investigate how honey affects gene expression profiling, paying particular attention to human intervention trials – ideally, large-scale randomized placebo-controlled studies – to gain valuable insights into their therapeutic and preventative applications and to create practical plans for reducing chronic inflammatory diseases [15].

The role of honey has been proven in various pathogenesis [16], and has therapeutic importance in Chinese and Unani medicine. The role of honey in health management has been discussed in various religious books [17,18,19]. Honey contains numerous substances, such as fructose, glucose, vitamins, amino acids, water, minerals, and enzymes [20,21], and it is interesting to note that certain ingredients have been found to play a role in disease cure. Honey has been shown to have antioxidant and anti-inflammatory properties, which make it effective for disease prevention and treatment. A combination of honey and ginger has been reported to reduce malondialdehyde (MDA) levels, making it a promising treatment remedy [22]. Honey facilitates a hypoglycemic effect and reduces oxidative stress, and its hepatoprotective role has been confirmed through the reduction in liver function enzyme activity [23,24]. Its role in cancer management has been confirmed in previous findings [25,26,27]. Honey has also been found to play a potential role in cancer management. This is due to its ability to modulate several biological activities, including apoptosis, angiogenesis, cell cycle, P13K/AKT, and signal transducer and activator of transcription 3 (STAT3). It has also been found to have a synergistic effect with drugs and other natural compounds, thereby improving their potency.

Oxygen-containing molecules known as reactive oxygen species (ROS) are created naturally as a consequence of metabolism. Oxidative stress is characterized by an imbalance between pro-oxidants and antioxidants, leading to dysregulation of redox circuits and damage to macromolecules [28]. The chemistry of ROS, reactive lipids, species reactive nitrogen species (RNS), and free radicals are involved in oxidative stress [29]. Antioxidant enzymes including superoxide dismutase (SOD), glutathione peroxidase (GPx), small molecular weight antioxidants like vitamins C and E, flavonoids, carotenoids, melatonin, ergothioneine, etc., work together to reduce the negative effects of oxidative stress [30].

Depending on the phase and context, ROS can both stimulate and suppress nuclear factor kappa B (NF-κB) signaling. In the context of oxidative stress, the NF-κB pathway can play both pro- and antioxidant roles. By suppressing ROS formation, promoting autophagy, inhibiting Jun N-terminal kinase activity, and enhancing antioxidant targets, the NF-κB pathway may provide protection in the context of oxidative stress [31]. In inflammatory tissues, cyclooxygenase-2 (COX-2) mediates pain, inflammation, and some catabolic responses. Cox-2 expression in the bovine synovial fibroblasts is directly correlated with oxidative stress. ROS could activate Cox-2 expression by activating NF-κB and extracellular signal-regulated kinase 1/2 (ERK1/2) in macrophages [32]. Induction of COX-2, inducible nitric oxide synthase (iNOS), abnormal expression of inflammatory cytokines (tumor necrosis factor [TNF], interleukin-1 [IL-1], IL-6), and chemokines (IL-8; CXC chemokine receptor 4), as well as variations in the expression of specific microRNAs, have also been described to play a role in oxidative stress-induced inflammation [33].

This review's main objectives are to highlight honey's potential medical benefits. In this context, the health advantages of honey and its constituents are reviewed, as well as the roles it plays in the treatment of different illnesses. Several sections and subsections accomplish a variety of study objectives, such as discussing its mechanism of action, possible involvement in managing different diseases, implications for modulating cell signaling pathways, and synergistic effects are discussed.

## 2 Active ingredients of honey

**Figure 1:**
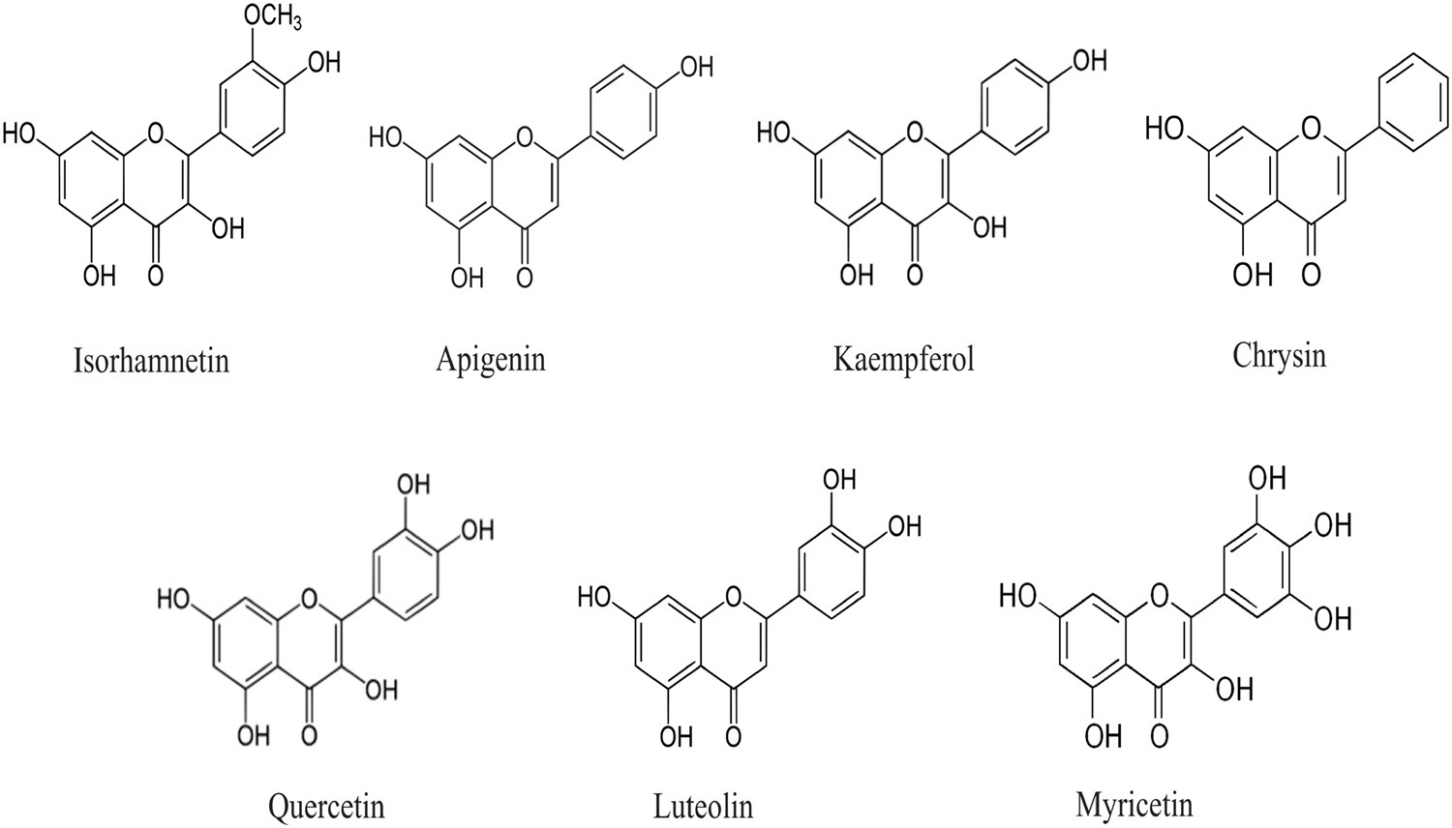
Chemical structure of flavonoids/polyphenols present in honey.

The botanical source of nectar from which honey is extracted determines its composition and quality, but other factors include geographic location, seasonal and climatic circumstances, processing method, and storage [34]. Honey has various valuable constituents, and such types of ingredients possess a significant role in health management through the modulation of various biological activities. It is interesting to note that honey is composed of over 180 different components, including amino acids, vitamins, minerals, and enzymes [35]. Additionally, this substance contains a variety of other valuable ingredients such as inhibin, proteins, phenols, antioxidants, and micronutrients [36]. It is impressive how many beneficial components can be found in a single substance. The other valuable ingredients are antibiotic-rich inhibin, proteins, phenols, antioxidants, and micronutrients. The chief enzymes of honey are diastase, invertase (saccharase), glucose oxidase [37], and vitamins like vitamins C, B (thiamine), B2 complex, and B6 (pantothenic acid) [19,37]. Honey also contains fructose and glucose as its main carbohydrate sources. Fructose is the main carbohydrate, followed by glucose at 28–40% and 20–35%, whereas the concentrations of disaccharides and trisaccharide’s are 1%, respectively [38]. Amino acids of physiological importance include glutamic acid, arginine, aspartic acid, proline, and cysteine [39], and polyphenols are also found in honey (Figure 1). Polyphenols in honey are mainly flavonoids, phenolic acids, and their derivatives, which provide honey with anti-bacterial and antioxidant potential [20].

The polyphenols in honey have direct roles in lowering or eliminating the risk of serious chronic human diseases. The beneficial flavonoid and phenolic acid components in honey are identical between varieties, but their relative amounts change. The known antioxidant properties of flavonoids or phenolic acids, which are found in trace amounts in nature, are the primary source of honey's therapeutic benefits [40]. Apigenin, catechin, chrysin, galangin, genistein, isorhamnetin, kaempferol, luteolin, myricetin, quercetin, rutin, pinocembrin, etc., are important polyphenolic compounds of honey [41].

Kaempferol has been shown to have protective effects against cardiovascular disease (CVD) through three general mechanisms: oxidative stress reduction, Ca^2+^-activated K^+^ channel activation and increased endothelial NOS activity by stimulating arterial relaxation, and suppression of TNF-α production and activation of NF-κB. It has also been discovered that kaempferol inhibits cyclooxygenase expression [42]. Pinocembrin, another flavonoid included in honey, has also been shown to possess a number of anti-inflammatory, antibacterial, and antioxidant qualities [43]. As per the United States Department of Agriculture database, varied types of honey samples from numerous countries hold isorhamnetin (0.06 mg/100 g), apigenin (0.03 mg/100 g), kaempferol (0.06 mg/100 g), quercetin (0.31 mg/100 g), luteolin (0.28 mg/100 g), as well as myricetin (0.36 mg/100 g) [44].

## 3 Mechanism of action of honey in the inhibition of pathogenesis

Honey has been found to have many health benefits because of its ability to regulate various biological activities. It inhibits pathogenesis by regulating oxidative stress, inflammation, and cell signaling pathways. This makes it a natural remedy for many health issues. It is fascinating to learn about the potential benefits of honey and its impact on various biological processes. Additionally, the health-promoting properties of honey could contribute to its ability to inhibit pathogenesis. The role of honey in disease management are described through different mechanisms of action as follows:

- One of the ways in which honey inhibits pathogenesis is through the regulation of oxidative stress. Oxidative stress is an imbalance between the generation and accumulation of ROS and antioxidants [45]. Under pathological conditions, free radicals are produced by both endogenous and exogenous systems. Honey has been found to have numerous pharmacological activities through the regulation of oxidative stress via the inhibition of pro-antioxidant agents and the enhancement of antioxidant enzyme levels. Honey is rich in phenols, which are recognized to scavenge and remove ROS [46], and are known to have antioxidant potential, which may modulate the production of free radicals [47]. Its role in different pathogeneses has been observed mainly through its antioxidant activity. A study was performed to characterize the phenolic acids, flavonoids, and antioxidant properties of monofloral honey collected from five different districts in Bangladesh. It was reported that a total of five different phenolic acids were found, with the most significant being caffeic acid, benzoic acid, gallic acid, followed by chlorogenic acid as well as trans-cinnamic acid. The flavonoids, catechin and kaempferol, were most abundant, followed by naringenin and myricetin. This funding confirmed that all examined honey samples are good sources of phenolic acids and flavonoids with good antioxidant properties [48].
- Krisnanda examined the impact of honey supplementation on male Wistar rats engaged in moderate-intensity physical activity. After a week, the plasma level of one of the key indicators of oxidative stress, MDA, was decreased in the group of rats that were given honey at a dosage of 5 g/kg body weight once daily [49]. Flavonoids in honey serve as protective agents against oxidative damage in human red blood cells [50]. The honey’s phenolic compounds, among others, take part in the regulation of the expression of genes involved in the reduction in oxidative stress, in the development of amyloid fibrils, as well as in elevating the expression of the Nrf2, which is accountable for the induction of antioxidant genes [51]. Honey contains various types of phenolic acids, with gallic acid being among the most significant for food due to its strong antioxidant properties. Additionally, other phenolic acids present in honey also exhibit antioxidant and antibacterial effects [52–56].
- The mechanisms of inflammation caused by different triggers are diverse and are not fully understood [57]. Preventing the production of inflammatory cytokines and mediators is vital for the regulation of inflammation. Honey and its bioactive compounds have been shown to have noteworthy anti-inflammatory properties by preventing the production of inflammatory cytokines and mediators. Honey regulates inflammatory cytokines and enzymes and inhibits pathogenesis. It may also inhibit chronic inflammation and oxidative stress as well as their related gene expression [58]. The expression of pro-inflammatory enzymes, including COX, which is involved in the metabolism of arachidonic acid and the production of prostaglandins (PG) and iNOS, which catalyze the production of NO, was also found to be decreased [59]. Honey polyphenols might function as agonists of NF-κB receptors and Toll-Like 4 receptors, both of which play a role in triggering inflammation and oxidative stress [15,60,61]. Moreover, quercetin honey flavonoid was confirmed as a PLA2 inhibitor in a study performed with human neutrophils [62]. Myricetin was explored by Lee and Lee [63] as inhibitor of the production of inflammatory mediators in keratinocytes treated with TNF-α. Kaempferol and acacetin were examined by Jiang et al. [64], who reported that they inhibited NO production by 50% at 29 and 7.7 µM, respectively.
- Honey and its bioactive compounds have been found to have a significant impact on the management of various types of cancer. This is achieved through the modulation of tumor suppressor genes, inflammation, cell cycle arrest, apoptosis, angiogenesis, and other cell signaling pathways. A recent study on lung cancer reported that Tualang honey upregulates pro-apoptotic (Bax, Bid, cytochrome c, SMAC), tumor suppressor (p53), caspases (caspase-3-8), cell surface death receptor (Fas), and cell cycle regulator (p21), and decreases the expression of two anti-apoptotic proteins (Bcl-w and B-cell lymphoma 2 [Bcl-2]) [65]. Sidr honey had a positive effect on the G1 phase proportions of cells, while decreasing the proportion of S and G2M [66]. Additionally, the water-soluble derivative of propolis appears to have the potential to prevent angiogenesis [67].
- Honey has been found to play a significant role in inhibiting the growth of microorganisms, which ultimately helps prevent microbial pathogenesis. Honey inhibits microbial growth by damaging the integrity of bacterial cell membranes and causing cell death. Moreover, the antibacterial potential of honey may be attributed to its osmotic activity, higher sugar content, lower moisture content, and the presence of gluconic acid, which causes antiseptic hydrogen peroxide (H_2_O_2_) [68]. Moreover, the anti-microbial mechanism of action showed that honey significantly obstructed the bacterial membrane’s permeability and increased potassium and protein leakage rates. Manuka honey (MH) has been shown to inhibit bacterial protein synthesis and cause bacterial DNA damage [69].

## 4 Role of honey in management of different diseases

### 4.1 Antioxidant activity

**Figure 2:**
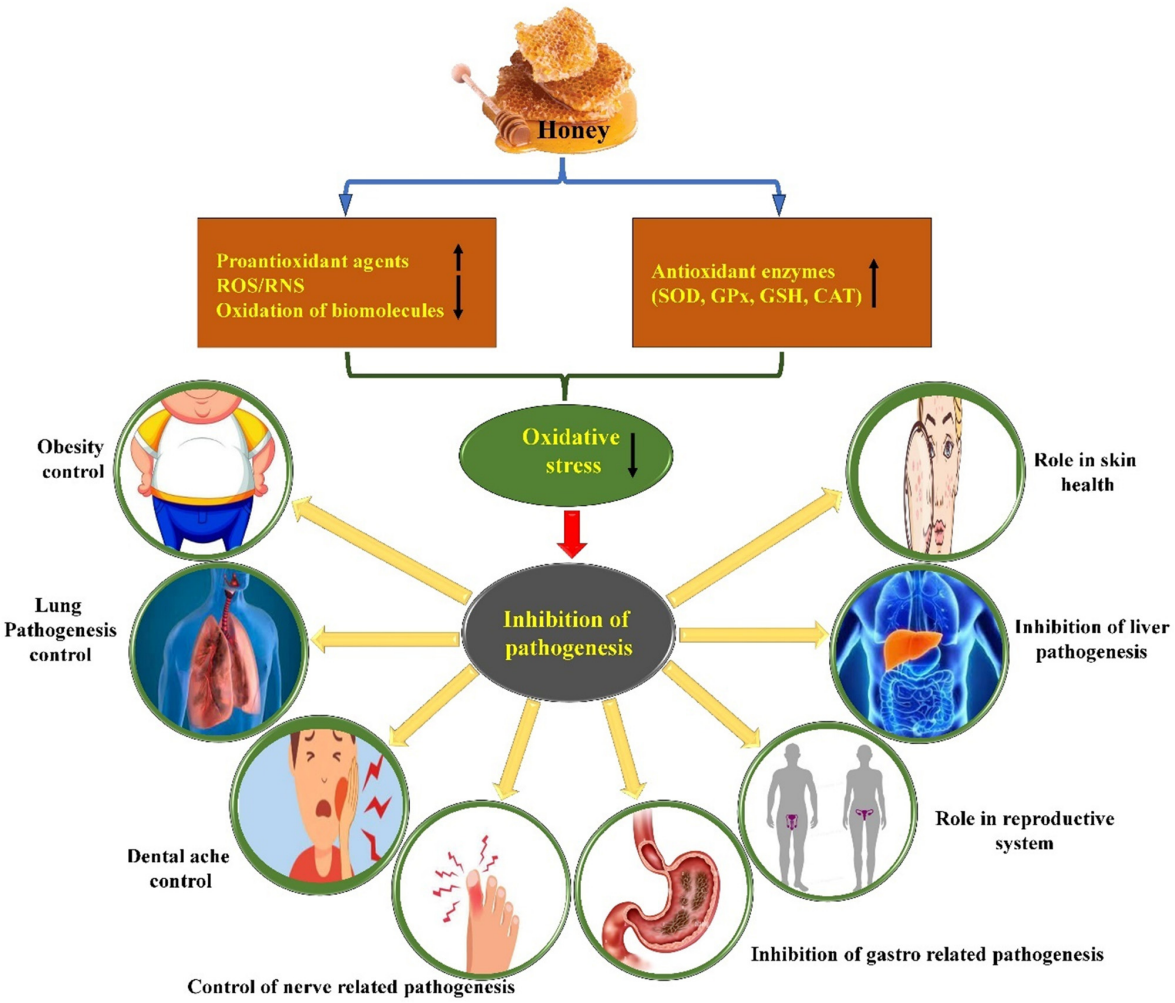
Honey shows role in different pathogenesis through the inhibition of pro-oxidant agents (ROS and RNS) and enhancement of antioxidant enzymes (SOD, catalase [CAT], Glutathione peroxidase [GPx], glutathione [GSH]).

Oxidative stress can lead to damage of cells, proteins, and DNA, contributing to various health issues. This can lead to cell damage and other negative effects owing to excessive oxidation [70]. Enhancement of antioxidant activity is of prime interest for the inhibition of pathogenesis. Various plants, as a whole or their parts such as fruits, stems, bark, seeds, and leaves, are potential sources of antioxidants that play a crucial role in neutralizing the effects of free radicals, minimizing intracellular oxidative stress, and ultimately preventing pathogenesis. From a health management perspective, honey plays a significant role in disease management through the inhibition of pro-antioxidant/oxidative agents and enhancement of antioxidant enzyme activities (Figure 2).

After the experiment, parsley and rhododendron honey displayed the most significant results in relation to the amount of phenolic compounds and antioxidant properties. Citrus and acacia honey samples showed the lowest antioxidant activities. These findings revealed that all honey samples had antioxidant effects [71]. Moreover, another study was performed to measure the antioxidant potential and quantify flavonoids and phenolics relating to quercetin and rutin in a few monofloral honeys. Flavonoids (rutin and quercetin), 2,2-diphenyl-1-picrylhydrazyl (DPPH), and total phenolic contents were used to measure the antioxidant properties of the honey samples. In terms of antioxidant activity, honey with the best EC_50_ consequence was A6-Aroeira. Significant differences were found between the antioxidant activities of the honey samples [72].

A study based on diabetic rats revealed that a mixture of glibenclamide and honey administered to diabetic rats showed increased CAT activity, decreased MDA levels, and decreased blood glucose levels [73]. Honey has been shown to have potential benefits in reducing hyperglycemia, improving oxidative stress, and slowing diabetic renal damage [74]. Moreover, Tualang honey supplementation showed a protective role against oxidative stress in the brain and anxiety-like behavior [75].

Experiments have been performed to assess the protective role of acetone in kidney and liver diseases induced by carbon tetrachloride (CCl_4_) in animal models. In addition, a combination of honey and silymarin pre-treatment prior to CCl_4_ intoxication significantly retarded the rate of increase in the serum levels of some enzyme markers as well as minimized oxidative stress [76]. A study evaluated the antibacterial and antioxidant effects of ginger, honey, and their blends, and the results revealed that a combination of both treatments resulted in a reduction in SOD and CAT activities. Furthermore, MDA and GSH levels were significantly elevated [22].

Another study investigated the antioxidant potential of honey in terms of free radical scavenging, bleaching inhibition, and reducing power. The findings showed that the antioxidant potential of this natural product correlated with the presence of total phenols, flavonoids, and water-miscible vitamins [77]. The phenolic acid and flavonoid contents of various types of Malaysian honey were evaluated, and it was found that different honey extracts possessed rich phenolic and flavonoid contents [78]. Furthermore, different experiments have revealed that Tualang honey contains the highest contents of phenolics, flavonoids, and DPPH radical-trapping activities among other Malaysian honey brands [78].

The antioxidant potential of honey varies among different brands, and a study conducted on different Malaysian honey types showed that sourwood honey and Longan honey varieties are superior antioxidant sources in comparison to MH [79]. Similarly, the free radical scavenging potential of the different honey varieties and Taulang honey showed the highest free radical scavenging potential.

### 4.2 Anti-inflammatory potential

**Figure 3:**
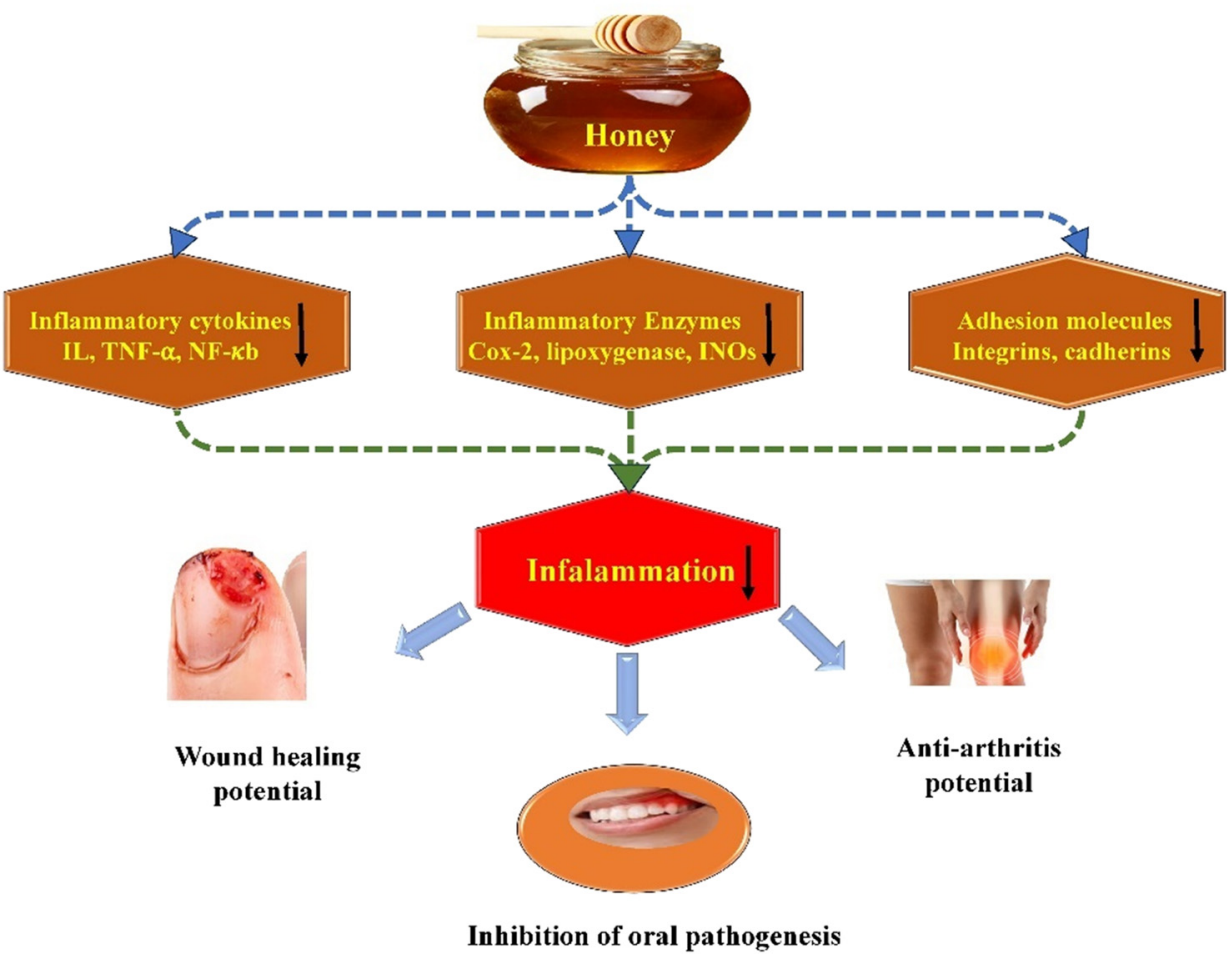
Honey and its bioactive compounds have been found to exhibit anti-inflammatory potential by inhibiting pro-inflammatory cytokines and enzymes (IL, TNF-α, NF-kB, COX).

Various vasoactive substances, including histamine, lymphokines, serotonin, prostanoids, chemokines, cytokines, and transcription factors that are typically produced in tissues, play a significant role in the inflammatory process [80]. Several medicinal plants have anti-inflammatory potential, which aids in wound healing. Honey, through the modulation of pro-inflammatory cytokines and enzymes, exhibits anti-inflammatory and wound-healing properties (Figure 3).

It is interesting to note that honey has been found to have anti-inflammatory properties, which may be attributed to its ability to regulate genes involved in this process. The protective role of the honey flavonoid extract on the synthesis of pro-inflammatory modulators was investigated, and the results showed that the extract significantly reduced pro-inflammatory cytokine release [81]. Furthermore, iNOS expression and reactive oxygen intermediate production are significantly inhibited [46,81]. Different varieties of honey, such as Manuka, Pasture, and Jelly bush, significantly augmented TNF-α, IL-1β, and IL-6 production in MM6 cells compared with raw and artificial honey-exposed cells [82]. The anti-inflammatory effects of honey in inflammation-induced rats have been previously described. According to the results, honey was found to reduce edema in inflamed rat paws and diminish the synthesis of plasma IL-6, nitric acid (NO), and TNF-α. Additionally, it was found to subdue COX-2, TNF-α, and IL-6 expression in paw tissue [83]. Greek honey samples showed anti-bacterial and anti-inflammatory properties, as evidenced by the diminished production of pro-inflammatory mediators [84]. Role of natural honey was documented in colitis and downregulation of oxidative and inflammatory markers [85].

Gelam honey reduced edema in inflamed rat paws in a dose-dependent manner. Additionally, gelam honey decreased the production of certain inflammatory markers such as NO, prostaglandin E2 (PEG2), IL-6, and TNF-α in plasma and decreased the expression of COX-2, iNOS, TNF-α, and IL-6 in paw tissue. Oral administration of gelam honey exhibited meaningfully decreased production of pro-inflammatory cytokines [83]. Kassim et al. reported that honey and its extracts were capable of preventing edema and pain in inflammatory tissues and demonstrated effective inhibitory potential against PGE and NO in both models. Reduction in pain and edema is associated with the inhibition of PGE and NO [86].

### 4.3 Wound healing effect

Wound healing is a complex process with various interdependent immunological and pathophysiological mediators to restore the cellular integrity of the damaged tissue [87]. The wound-healing power of honey has been recognized as a natural healing process [88] and could be used as an ideal dressing in the handling of burns [89]. Wounds exposed to topical use of honey showed less edema, few polymorphonuclear lymphocytes, minimal necrosis, improved wound contraction, better epithelialization, and healthier tissue organization [90]. The anti-inflammatory mechanism of honey based on the rat model was examined, and the findings of the experiment demonstrated its inhibitory effects by attenuating the translocation of NF-κB to the nucleus and inhibition of inhibitor of nuclear factor kappa B (IκBα) degradation, with a successive decrease in inflammatory mediators [91].

Moreover, honey on airway tissues was examined, and it was reported that treatment with aerosolized honey reduced the airway inflammatory cell number and inhibited goblet cell hyperplasia [92]. An important study has proven the role of MH in wound healing [93]. The wound-healing effect of honey was examined using a combination of topical honey with silver nanoparticles, and the findings revealed that multiflora honey with silver nanoparticles improved the efficiency of wound contraction compared to honey only [94]. Another recent finding revealed that topical application of honey played a role in wound contraction on postoperative day 9 in both the diabetic and nondiabetic groups [95].

### 4.4 Anti-tumor activity

**Figure 4:**
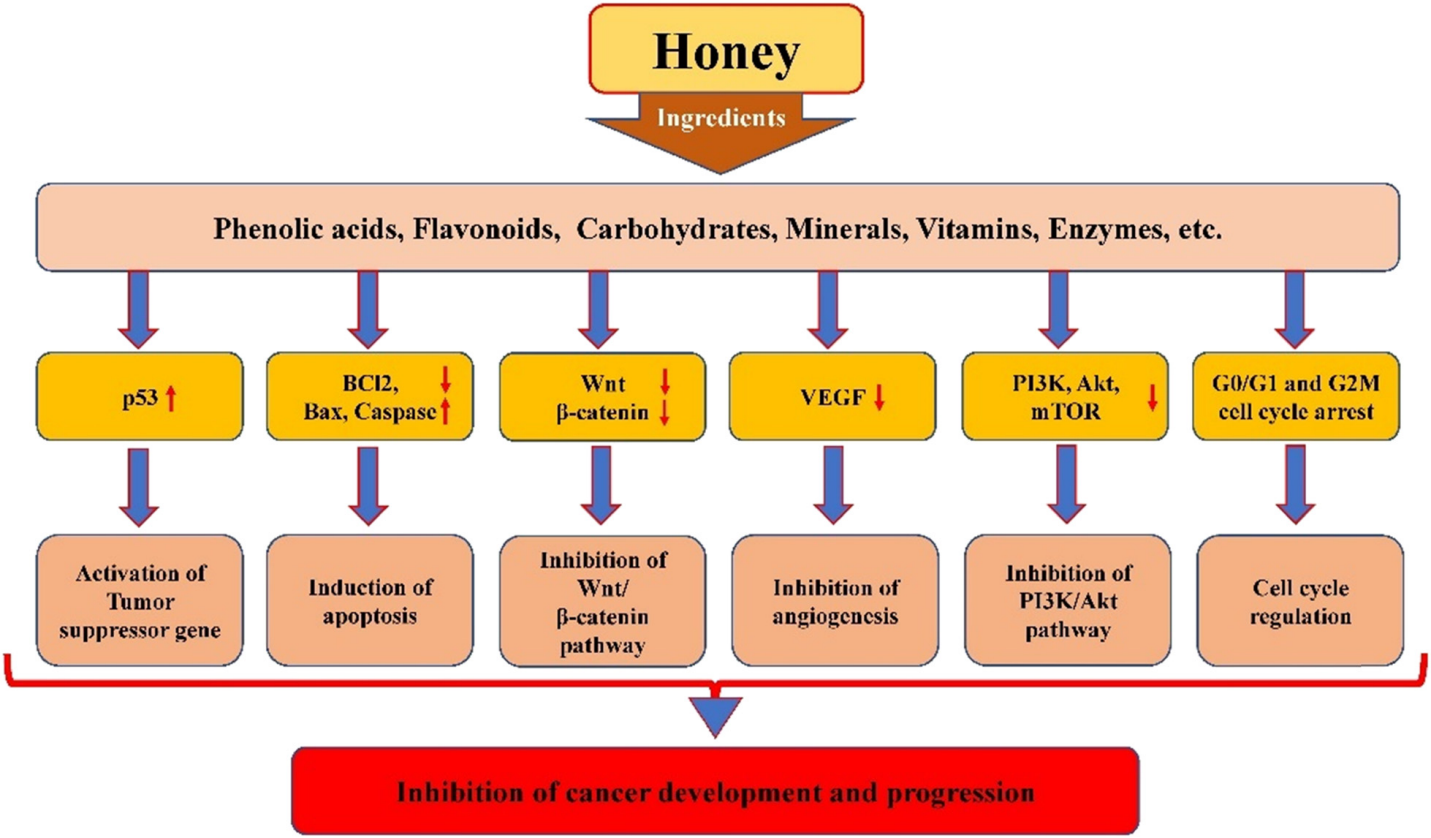
Role of honey in the management of cancer by the regulation of different cell signaling pathways.

Cancer is a complex disease that can be triggered by changes in gene structure and function. Radiation and chemotherapy are commonly used in the treatment of cancer, but they can have negative side effects and even cause changes in normal genes. Although these treatments are effective, they can lead to secondary complications. A considerable number of literature reviews imply that several natural products have inhibitory activities against cancer development and progression [96–101], without any adverse side effects. Honey and its important ingredients play an effective role in cancer management through modulation of cell signaling pathways (Figure 4). Some experiments were conducted to evaluate the effect of honey treatment on the development of breast cancer in animal models, and the findings of such studies revealed that breast cancers have a slower growth rate and slight mean tumor size in honey-treated groups as compared to disease controls [102]. These findings were further supported histologically, as honey-treated cancers had scores of 1 and 2 as compared to grade 3 observed in disease control groups [102].

The role of chrysin and honey has also been evaluated in murine and human melanoma cell lines for their anti-proliferative activities, and the results of the study showed that both these compounds possess anti-proliferative potential on melanoma cells in a concentration-as well as duration-dependent manner [103]. Previous studies have evaluated the anti-proliferative potential of MH on different cancer cell lines (murine melanoma, human breast cancer cells, and colorectal carcinoma), and the results revealed that honey possesses a potent anti-proliferative effect on all cancer cell lines in a duration- and concentration-dependent manner [104]. Furthermore, dietary supplementation with honey and black seed has shown a protective effect against oxidative stress, inflammatory response, and carcinogenesis in animal models [105].

Numerous studies have confirmed that honey plays an effective role in the management of cancer by regulating various cell signaling pathways, as discussed here.

#### 4.4.1 Tumor suppressor gene

Tumor formation is closely linked to the prevention of programmed cell death, or apoptosis, which confers cell longevity. Tumors have been shown to exhibit angiogenesis and angiogenic factors, which may be important for the creation and growth of tumors. Tumor-suppressor genes help cells develop normally while preventing the spread of cancer [106]. Tumor suppressor genes are crucial factors in preventing the development and progression of tumors. One of the most well-known tumor suppressors is p53, often referred to as the “guardian of genes.” It plays a critical role in connecting various cell signaling pathways to help regulate cell growth and division. Altered expression of this protein has been observed in cancer cells [107]. Natural products and their constituents play a potential role in the enhancement of p53 gene activity and ultimately control tumor development and progression. A study was performed to assess the function of honey in the induction of apoptosis and the underlying molecular mechanisms in colon cancer cell growth inhibition. The results clearly showed that honey transduces apoptotic signals, induces apoptosis by upregulating the p53 gene, and modulates the expression of pro- and anti-apoptotic proteins [108] (Table 2).

#### 4.4.2 Apoptosis

It is understood that apoptosis is a crucial mechanism for development as well as tightly regulated cell death [109]. Natural products or bioactive molecules, including honey, have been shown to be implicated in cancer management through the induction of apoptosis. The use of honey has been studied to determine its role in human renal cancer cell lines and to investigate its anti-proliferative, apoptotic, and antitumor activities. Honey decreases malignant cell viability in a dose- and duration-dependent manner and induces cancer cell apoptosis in a dose-dependent way [110]. The apoptotic and anti-proliferative roles of different honey types have been studied in human leukemia cells, and the results have indicated that crude commercial honey types induce apoptosis in a dose- and duration-dependent manner [111] (Table 1).

**Table 1:** Role of honey in tumor prevention through modulation of cell signaling pathways

Pathways	Cancer	Cell line	Mechanism	Refs.
Tumor suppressor gene	Colon cancer	HCT-15 and HT-29	Honey-initiated apoptosis was followed by up-regulating p53 to modulate the expression of pro and anti-apoptotic proteins	[108]
Apoptosis	Renal cancer	ACHN	Honey provoked apoptosis of the ACHN cells	[110]
	Leukemia	HL-60	Honey induced apoptosis in HL-60 cells based on ROS-independent cell death pathway	[111]
	Breast cancer	MCF-7 and MDA-MB-231	*Tualang* honey meaningfully increased the % of apoptotic cells in both the ER*α*-negative and ER*α*-positive breast cancer cells in a time-dependent way	[112]
	Breast and cervix cancer	MCF-7 and MDA-MB-231 and HeLa	*Tualang* honey decreases the mitochondrial membrane potential in the cancer cell lines. The activation of caspase-3/7 and -9 was noticed in all *Tualang* honey-treated cancer cells	[113]
	Oral squamous cell carcinomas and human osteosarcoma	OSCC and HOS	Early apoptosis was obvious where % of early apoptotic cells enhanced in dose and time-dependent way	[114]
	Colon cancer	HT29	Both Gelam as well as Nenas honeys induced apoptosis meaningfully	[115]
Angiogenesis	Breast cancer	MCF7	Malaysian Tualang honey meaningfully reduced VEGF secretion in breast cancer cells	[121]
mTOR	Colon cancer	HT29	Honey and ginger could potentially be used as a therapy for colorectal cancer by preventing mTOR and Wnt/β catenin signaling pathways	[123]
AKT	Colon cancer	HT29	The combination of both ginger as well as Gelam honey produced higher downregulation of *AKT* gene expression at a greatly lower concentration as compared to either treatment only	[124]
Cell cycle	Lung cancer	H23 and A549	Accumulation of Tualang honey treated adenocarcinoma cells in sub-G_1_ and G_2_/M phases	[65]
	Melanoma	A375 and B16-F1	Cytotoxicity initiated by honey or chrysin was arbitrated by G (0)/G (1) cell cycle arrest	[103]
	Colon cancer	HCT-116 and LoVo	MH-induced cell cycle arrest in the S phase in cancer cells, it occurred in the G2/M phase via the modulation of cell cycle regulator genes	[126]
STAT3	Breast cancer	MCF-7 and MDA-MB-231	MH caused a fast reduction in tyrosine-phosphorylated STAT3	[129]
	Lung and breast cancer	A549 and MDA-MB-231	MH-mediated inhibition of p-STAT3 in lung cancer and breast cancer	[130]
Wnt/β-catenin	Colon cancer	HT29	Gelam honey shows apoptosis in a dose-dependent means and dose-dependent expressions of β-catenin, Akt, mTOR	[123]

In addition, honey has been found to modulate tamoxifen activity through direct induction of caspase-dependent apoptosis. The combination of honey and tamoxifen has a greater inhibitory effect on both estrogen receptor-negative and estrogen receptor-positive breast cancer cells [112] (Table 2). The anti-cancer role of Tualang honey has been evaluated in different cell lines, such as human breast cancer, cell lines of cervical cancer, and breast epithelial normal cell lines. An enhancement in lactate dehydrogenase (LDH) leakage from the cancer cell membranes indicates that honey possesses cytotoxicity against all cancer cells, but this effect was not found in normal breast epithelial cells. It was also noted that activation of caspase-3, -7, and -9 occurred in all cells treated with honey [113] (Table 1).

**Table 2:** Potential role of honey in disease prevention

Potential action	Outcome of the study	Refs.
Antioxidant potential	A combination of glibenclamide and honey improved CAT activity, and diminished MDA levels	[73]
	Tualang honey supplementation showed a protective role against oxidative stress in the brain and anxiety-like behavior	[75]
	The antioxidant potential of the honey types was in correlation with their biochemical constituents like total phenols, flavonoids, and water-miscible vitamins	[77]
	Tualang honey shows antioxidant potential	[78]
	The free radical scavenging potential of different honey varieties and Taulang honey shows the uppermost free radical scavenging potential	[45]
Anti-inflammatory potential	Different varieties of honey significantly augmented the TNF-α, IL-1β, and IL-6 production from MM6 cells	[82]
	Honey reduces the synthesis of NO, TNF-α, and IL-6 in plasma, and subdues COX-2, TNF-α, and IL-6 expression	[83]
Wound healing activity	Honey showed noteworthy role in natural healing process	[85]
	Wounds exposed with a topical use of honey showed lesser edema, better wound contraction, better epithelialization, and healthier tissue organization	[86]
Anti-bacterial effects	Honey shows anti-bacterial potential against tested organisms while *Salmonella typhi, Escherichia coli* (*E. coli*), and *Pseudomonas aeruginosa* (*P. aeruginosa*) displayed sensibility toward all these extracts	[137]
	Most of the bacteria were inhibited by all honey varieties	[138]
	The anti-bacterial potential was confirmed by the diameter of the inhibition zone of honey for *E. coli* and *P. aeruginosa*, which was 0–30 and 0–38 mm, respectively	[139]
Anti-viral activity	Honey showed anti-viral activity against *Varicella zoster*	[142]
	Honey powerfully inhibits the replication of the influenza virus, so it is directly associated with its virucidal effects	[143]
Anti-fungal activity	Varied concentrations of honey were used against yeast, and it was observed that all of the yeast strains were inhibited	[147]
	Flavonoid extract can block the dimorphic conversion of *C.* albicans	[51]
	Honey samples showed variable levels of inhibitory activities at various concentrations against the tested fungi	[148]
Immunomodulatory activity	Honey use may drastically increase the production of interleukins like IL-1β as well as IL-6 in infected mice and improve macrophage-killing	[149]
	Manuka, Pasture, Nigerian Jungle, and Royal Jelly showed immunomodulatory effects	[25,150,151]
	Niger oligosaccharides are present in honey which has been reported to have immune potentiating	[152]
	Gelam honey minimizes the production of various inflammatory protein expression	[153]
Hepatoprotective activity	Animals, that were pre-treated with silymarin and honey before paracetamol administration, significantly resisted the increase in serum level of hepatic function marker enzyme	[155]
	Honey (high dose) meaningfully improves liver damage and decreases the liver function enzymes	[156]
	Honey establishes important hepatoprotective activities by decreasing the liver marker enzymes	[157]
	Thyme honey improves hepatic complications and decreases the rate of liver cell damage	[158]
Neuroprotective activity	The use of honey mitigates overall anxiety and showed role as neuroprotective agent	[162]
	Honey potentially decreased locomotion and rearing behaviors	[163]
	Pre-treatment with Tualang honey reduced neuronal degeneration	[165]
	Neurotoxicity initiated by exposure to AlCl_3_ corroborated the antioxidant and neuroprotective effect of chrysin	[167]
Anti-diabetic activity	A combination of glibenclamide or metformin with honey showed anti-diabetic potential	[169]
	Honey meaningfully reduced TGs as well as VLDL cholesterol	[170]
Cardioprotective effects	Administration of honey protects the heart by possessing antiarrhythmic activity, reducing the infarction size	[180]
	The honey administration also significantly decreases the sum of ventricular ectopic beats and lowers the frequency of reversible ventricular fibrillation	[181]
	Incidence as well as duration of reversible ventricular fibrillation were decreased by honey	[181]
	Honey showed an important decrease in the incidences of ventricular tachycardia	[183]
Effects on dental health	Honey has a powerful remedial potential in the management of gingivitis and periodontal ailments	[187]
	MH was able to reduce bleeding and the amount of plaque in patients with gingivitis and plaque	[188]
	Decrease in plaque indices in the honey group as well as chlorhexidine + xylitol group when compared to chlorhexidine	[190]
Effects on eye health	Honey meaningfully decreases total colony-forming units for the eyelids	[191]
	Application of honey in the injured corneas showed quicker healing of epithelial	[194]
	Topical honey eye drops, when used along with fluorometholone and cromolyn eye drops, might be useful for the treatment of vernal keratoconjunctivitis	[194]
Renoprotective effects	Tualang honey supplementation showed a renoprotective effect	[198]
	Honey feeding meaningfully decreases cisplatin-induced tubular epithelial cell death, cytokine and chemokine expression, and immune infiltration into the kidney	[199]
	Honey treatment showed a role in the prevention of elevations in serum creatinine	[200]
Anti-hypertensive potential	Honey supplementation meaningfully reduced systolic blood pressure and MDA levels in SHR	[209]
	Honey has anti-prehypertension potential	[211]
Anti-allergy activity	Mast cell degranulation was suggestively inhibited by using honey. MH could be good remedy in the treatment of AD	[212]
	The honey mixture seems valuable in the managing of PV and dermatitis	[213]
	Consumption of honey presented a substantial improvement in individual allergic rhinitis symptoms	[214]
Effects on the respiratory system	Honey was used to efficiently treat asthma, and it might demonstrate to be a promising treatment in asthma	[92]
	A high dose of Gelam honey improves the histopathological changes in mice model of allergic asthma	[162]
	Treatment with honey improved the adverse effects noticed in the lungs and Tualang honey may defend against paraquat-caused toxicity in the rat lung	[217]
Effects on skin	Scaling was gone and itching was comforted within 1 week. Skin lesions were healed and gone completely within 2 weeks	[230]
	Honey in combination with glycerin compared to standard anti-bacterial soap treatment showed additional effectiveness	[231]
Reproductive activity	Malaysian honey taken daily meaningfully enhanced the count of epididymal sperm without affecting reproductive hormones and spermatid count	[234]
	Honey supplementation meaningly enhanced epididymis and testis weights	[236]
	Honey caused helpful effects in decreasing the increase in cortisol as well as in increasing the decrease in progesterone levels	[237]
Radioprotective activity	Kelulut honey was recognized to decrease body curvature potential in the irradiated embryos	[240]
	Genotoxic damage by X-radiation inhibited or alleviated by adding honey	[241]
	Supplementation of propolis with radiotherapy treatment shows a pretty measurable protection against DNA damage that began by ionizing radiation	[242]

The antiproliferative activity of Tualang honey on HOS and OSCC cell lines was investigated, and the results revealed that cell viability was based on the duration and concentration-dependent inhibitory effects of honey on these cell lines [114]. The chemopreventive activities of two honey types (gelam and Nenas Monofloral) have been investigated in colon tumor cells. Both honey types have the potential to suppress colon tumor growth through the induction of apoptosis and suppression of inflammation [115]. Another study was performed to check the influence of honey and *Aloe vera* on tumor growth, and the data of this study suggested that both natural products can decrease tumor growth by reducing cell proliferation and inducing apoptosis susceptibility [116]. The molecular mechanism of the anti-proliferative effect of acacia honey was investigated, and the results showed that honey inhibited cell proliferation, arrested the G0/G1 growth phase, stimulated cytokines, enhanced calcium ion release, and suppressed Bcl-2 and p53 gene expression in a concentration-dependent fashion [117]. An experiment was conducted to investigate the effects of chrysin and honey on prostate cancer cells. In this study, we aimed to determine the apoptotic and anti-proliferative potential of these compounds. The results showed that both chrysin and honey had anti-proliferative effects on prostate cancer cells. Moreover, the effect was concentration- and duration-dependent [118] (Table 1).

#### 4.4.3 Angiogenesis

Angiogenesis is indeed a crucial factor in tumor growth and progression, and vascular endothelial growth factor (VEGF) has been identified as an independent prognostic factor in patients with all stages of ovarian cancer [119]. Various medicinal plants have been confirmed to have beneficial roles in the inhibition of angiogenesis and prevention of tumor growth. A study based on gastric carcinoma cell lines showed that zerumbone also inhibited cell proliferation, VEGF expression, and NF-κB activation [120]. The anti-angiogenic effects of Malaysian Tualang honey were already explained. Effects of Malaysian Tualang Honey on VEGF-induced human umbilical vein endothelial cell (HUVEC) angiogenesis was evaluated. Moreover, matrix metalloproteinase-2 secretion from HUVEC and VEGF production from MCF7 cancer cells in response to honey were measured. Malaysian Tualang honey suppressed VEGF-induced HUVEC proliferation, migration, and tube. Malaysian Tualang honey significantly reduced VEGF secretion in breast cancer cells [121] (Table 1).

#### 4.4.4 mTOR and AkT pathway

Cells use the PI3K/AKT/mTOR pathway as a signaling route in response to growth factors, hormones, and nutrients. It is essential for cell growth, survival, and metabolism and is frequently changed in different types of cancer [122]. Laboratory studies have confirmed that medicinal plants and their components prevent tumor formation because they are rich sources of antioxidants and other valuable components. Honey has proven its potential role in tumor inhibition via the regulation or inhibition of the mTOR and AkT pathways. An experiment was performed to determine whether the combination of ginger and honey possesses chemopreventive potential in colon cancer cells through the regulation of mTOR, Wnt/β-catenin, and apoptosis signaling pathways. The potential benefits of the gelam honey and ginger combination in preventing colorectal cancer were noted. This combination may induce apoptosis and inhibit the Wnt/β-catenin and mammalian target of rapamycin pathway (mTOR pathways) [123]. The combination of honey and ginger extract has been described to have potential chemopreventive properties in cancer cells. According to a previous study, the combination treatment of ginger and honey resulted in cell death, which was found to be associated with the upregulation of IκB and caspase 9 genes, and a decrease in NF-kB AkT and Bcl-xL genes in a synergistic manner [124].

#### 4.4.5 Cell cycle

Controlling the cell cycle is an important step in cancer prevention. Honey has been shown to play a role in cancer management by regulating the cell cycle. A human lung adenocarcinoma cell-based study reported that cell cycle analysis verified the accumulation of Tualang honey-treated adenocarcinoma cells in sub-G_1_ and G_2_/M phases [65]. This study was conducted to investigate the anti-proliferative potential of honey and chrysin in human melanoma. This study found that both compounds were able to exert an anti-proliferative effect on cancer cells in a dose- and time-dependent manner. Moreover, the cytotoxicity initiated by honey or chrysin was mediated via G0/G1 cell cycle arrest and the initiation of hyperploid progression. This finding suggests that the anti-proliferative effects of honey are mostly due to the presence of chrysin [103] (Table 1).

Breast cancer-based study was conducted to investigate the effects of 1% Tualang honey on two different cell lines, MCF-7 and MDA-MB-231. The study found that treatment with 1% Tualang honey caused G2/M phase arrest in MCF-7 cells, whereas S phase arrest was observed in MDA-MB-231 cells. These findings suggest that Tualang honey can potentially be used as a treatment for breast cancer. This study demonstrated that Tualang honey has differential effects on cell cycle progression in MDA-MB-231 and MCF-7 cells, which are both types of breast cancer cells. The study found that these effects were mediated by p53-dependent and independent p21 signaling, respectively [125], and MH displayed deep inhibitory effects on cellular growth through induction of apoptosis [126] (Table 1).

#### 4.4.6 STAT3

One transcription factor that is activated after numerous important cytokine receptors that are produced by lymphocytes is STAT3. As a result, it is essential for controlling CD4+ and CD8+ T cells as well as B cells. There have been reported cases of patients with immunodeficiency and immunological dysregulation caused by loss-of-function or gain-of-function mutations in STAT3 [127]. STAT3 is the most frequently overexpressed or constitutively activated gene in nearly 70% of solid and hematological tumors [128] (Table 1). Aryappalli et al. reported that MH on MCF-7 and MDA-MB-231 cells, both of which are different types of breast cancer cells. The study found that MH induced apoptosis or programmed cell death in MCF-7 cells through the activation of caspases 9 and 6. Additionally, at low concentrations, MH caused a rapid reduction in tyrosine-phosphorylated STAT3 in both MCF-7 and MDA-MB-231 cells. Highest inhibition of pY-STAT3 was observed at 1 h, with a loss of more than 80%, and coincided with reduced IL-6 production. These findings recognize many functional pathways affected by honey in human breast cancer and highlight the IL-6/STAT3 signaling pathway as one of the initial potential targets [129] (Table 1). MH-mediated inhibition of p-STAT3 has been reported in lung and breast cancer cell lines [130].

#### 4.4.7 Wnt/β catenin

The Wnt/β-catenin pathway is made up of a family of proteins that are essential for both adult tissue homeostasis and embryonic development. Cancer and non-cancer disorders are among the many dangerous diseases that are frequently brought on by the dysregulation of Wnt/β-catenin signaling [131]. Experiments were conducted to evaluate whether the combination of honey and ginger might have chemopreventive potential in colon cancer cells by modulating the apoptosis and Wnt/β-catenin and mTOR signaling pathways. Gelam honey, ginger, and their combination induced apoptosis in a dose-dependent manner. Moreover, combined treatment decreased the gene expression of β-catenin, Akt, mTOR, and cyclin D1, whereas caspase-3 and cytochrome C genes were enhanced. The combination of gelam honey and ginger has been suggested as a potential therapy for colorectal cancer. This is due to their ability to inhibit the Wnt/β-catenin and mTOR signaling pathways, which are known to play a role in the development and progression of colorectal cancer [123]. The anti-proliferative effect of some Indian honey types and their mechanism of action in colon cancer were investigated. Indian honey samples exhibited a substantial inhibitory effect on cell growth by inducing apoptosis, preventing cell proliferation, and limiting the cell cycle to the G2/M phase in colon cancer cells. Moreover, honey samples were also shown to inhibit the β-catenin/Wnt pathway [132] (Table 1).

### 4.5 Anti-microbial activity

Multi-drug resistance of microbes is a serious challenge in the medical field, and infections caused by multi-drug-resistant bacteria, mainly in intensive care units, pose a severe problem [93,133]. The use of natural products from either animals or plants to prevent pathogenesis is of principal interest in health sciences. In this respect, highly active compounds have a potential role in the retardation of microorganism growth. An abundant literature survey regarding the valuable role of honey confirms its anti-bacterial, anti-fungal, and anti-parasitic properties, as described in Table 2. Sumra honey displayed a minimum inhibitory concentration (MIC) against the clinical isolates of *Staphylococcus aureus*. Sumra honey has shown promising results in inhibiting biofilm formation by various bacteria, including *S. aureus*, *Bacillus subtilis*, *P. aeruginosa*, and *E. coli*. Several studies have confirmed that honey plays a protective role in inhibiting microorganisms [134].

#### 4.5.1 Anti-bacterial activity

Although bacteria are essential to human health, they may also be the cause of a wide range of dangerous infections and diseases. The appropriateness of therapy can have a significant clinical impact because severe bacterial infections are known to have high rates of morbidity and mortality. These infections are severe because of the bacteria's virulence characteristics, the fact that they often occur in individuals who are already disabled, and their resistance to medications, which makes treating infections more difficult [135]. *Bacteria* are among the chief players in most diseases and deaths worldwide. Thus, honey plays a significant role in slowing the growth of microorganisms. Honey is known to be highly effective in inhibiting the growth of microorganisms. The precise mode of honey’s inhibitory action on bacterial growth is not completely understood. However, honey’s anti-microbial activity is proposed to be due to the release of H_2_O_2_ as the dilution of honey leads to the activation of glucose oxidase, which oxidizes glucose to gluconic acid plus H_2_O_2_ [136]. The ant-bacterial potential of various types of raw and processed honey extracts was evaluated, and experimental results have shown that both types of honey show anti-bacterial potential against the tested organisms [137].

**Figure 5:**
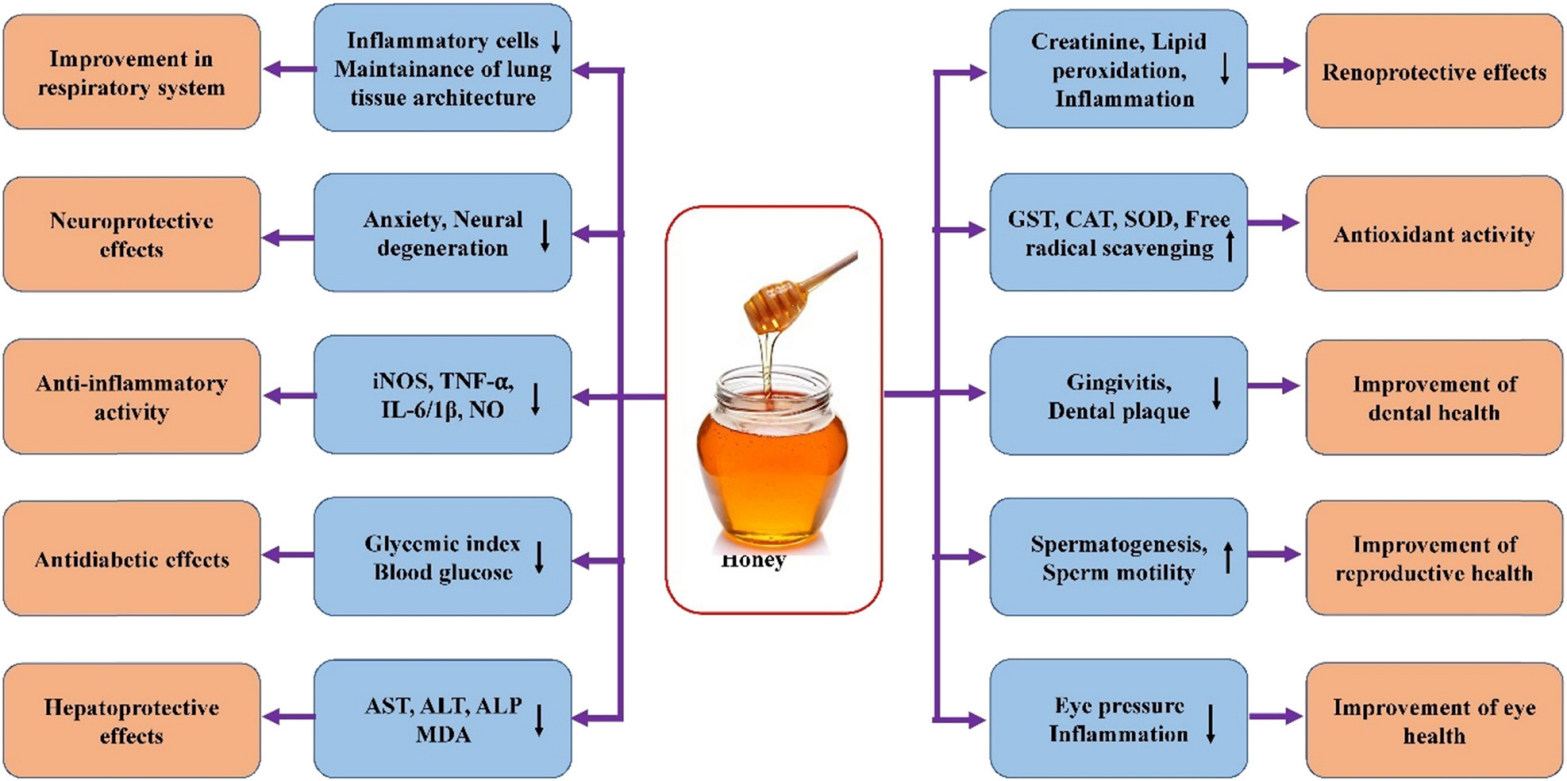
Honey may have a role in disease management by modulating various biological activities. iNOS, TNF-α, IL-6, NO, ALP, ALT, AST, Glutathione S-transferase (GST), SOD, CAT.

The anti-bacterial activities of locally produced honey and commercial therapeutic honey (Medi and Manuka) were evaluated and compared. The results showed that most of the bacteria (12 out of 13) were inhibited by all honey varieties, while only *Candida albicans* and *Serratia marcescens* were not inhibited [138]. Furthermore, among all types of honey, the Medi and MH types showed the best anti-bacterial effects [97,138] (Figure 5). In addition, the anti-bacterial potential of different honey types was evaluated against gram-negative organisms. The results confirmed that the diameters of the inhibition zones of honey against *E*. coli and *P. aeruginosa* were 0–30 and 0–38 mm, respectively [139].

#### 4.5.2 Anti-viral activity

People of all ages frequently get sick from viral infections. Although there is a wide range of illnesses, small children and people with weakened or compromised immune systems are more likely to suffer from severe sickness [140]. Worldwide, a diverse variety of viruses cause significant morbidity and mortality. Viral epidemics can be particularly destructive in nations with weak health systems and dysfunctional governments. Antiviral therapy is currently unavailable for the majority of these diseases. In order to contain these viruses, prevention through the use of efficient infection management and immunization is crucial [141]. Honey has also been used for its anti-viral activity and has shown promising effects against some viruses (Table 2). The anti-viral effects of honey (Manuka and Clover types) against *Varicella zoster* were examined. The results confirmed that these honey types showed anti-viral activity against *Varicella zoster* [142]. MH efficiently inhibited influenza virus replication, which is related to its virucidal effects [143].

#### 4.5.3 Anti-fungal activity

A major public health risk is fungal infections. Fungal infections are linked to mortality and potentially fatal mycoses in patients with various diseases, particularly COVID-19. Depending on their severity, fungal infections can be superficial, cutaneous, subcutaneous, mucosal, or systemic [144]. The high toxicity of the chemicals and the present small antifungal arsenal are linked to the high rates of morbidity and mortality brought on by fungal infections. Furthermore, because fungal and human cells share many characteristics, it can be difficult to find new therapeutic targets. However, fungi have evolved resistance strategies, such as biofilm formation and efflux pump protein overexpression, highlighting the significance of comprehending these mechanisms [145]. It is interesting to note that the anti-fungal properties of honey have been recognized since ancient times. It is good to know that researchers are still exploring this potential today. It is noteworthy that the honey types from Algeria were tested for their anti-fungal activities against pathogenic yeast, and the results showed that Rhodotorula sp. was susceptible to honey, whereas Candida albicans was resistant to all honey concentrations used [146].

However, in another study, various concentrations of honey were used against yeast, and it was observed that all yeast strains were inhibited [147]. A different study revealed that honey flavonoid extract can block the dimorphic conversion of *C. albicans* [51]. The anti-fungal activities of some honey samples from Nigeria were determined, and it has been confirmed that such honey samples showed variable levels of inhibitory activities at various concentrations against the tested fungi, with inhibition zones increasing with higher honey concentrations [148].

### 4.6 Immunomodulatory effects

The immunomodulatory activity of honey has been previously reported (Table 2). An important study explored the role of honey in innate immunity and survival rate during invasive aspergillosis. This finding demonstrated that honey use may considerably enhance the production of interleukins like IL-1β as well as IL-6 in infected mice and improve macrophage killing [149]. Honey brands such as Manuka, Pasture, Nigerian Jungle, and Royal Jelly have been found to increase Interlukin-1, Interlukin-6, and TNF-⍺ production [25,150,151]. Niger oligosaccharides, a mixture of nigerose and nigerosyl maltooligosaccharides, are present in honey and have been reported to have immune-potentiating potential [152]. Gelam honey might decrease edema in rat paws, reduce plasma NO production, TNF-α, and IL-6, and suppress iNOS expression, TNF-α, Cox-2, and IL-6 levels in paw tissue [153].

### 4.7 Hepatoprotective effects

Globally, liver disease causes about 2 million fatalities annually, with 1 million coming from cirrhosis-related complications, 1 million from viral hepatitis, and 1 million from hepatocellular carcinoma. One of the main causes of liver disease in the world is alcohol-associated liver disease [154]. Honey has been used by the general public as a general hepatoprotective agent since ancient times. In this regard, honey was evaluated in diabetic animal models to evaluate liver function enzymes. The outcomes of the study have revealed that it significantly reduces the elevated levels of aspartate aminotransferase (AST), alanine transaminase (ALT), and alkaline phosphatase (ALP) activities, thus acting as an efficient hepatoprotective agent in diabetes [24] (Figure 5, Table 2).

*A. cerana* honey has been found to have a positive effect on the liver. Honey has been shown to inhibit acute alcohol-induced increases in AST and alanine aminotransferase levels, reduce the production of hepatic MDA, and increase GPx and SOD activities. Additionally, it has been found to significantly inhibit the level of Transforming growth factor beta-1 (TGF-β1) in the serum and liver [155]. A high dose of *A. cerana* honey appears to have a positive effect on liver damage. According to a study, it has been shown to reduce serum alanine aminotransferase and AST levels, prevent the MDA content, and increase the activities of GPx and SOD. Additionally, it was found to lower the expression of TGF-β1, which was experimentally induced by bromobenzene [156]. In mice treated with CCl_4_, there was a significant increase in serum aminotransferases and ALP activity as well as a reduction in antioxidant enzymes and total antioxidant capacity. However, honey treatment has been found to have important hepatoprotective effects. It can decrease liver marker enzymes to normal levels and restore antioxidant enzyme levels [157].

The use of thyme honey appears to have a positive effect on the histopathological parameters of the liver tissue. According to a study, it has been shown to manage hypertrophic degeneration and nucleus changes, hepatic necrosis, inflammation, hypertrophy of Kupffer cells, enlargement of sinusoids, and fibrosis. Based on these findings, it seems that the high percentage of antioxidants in thyme honey improves hepatic complications and decreases the rate of hepatocellular damage [158]. The administration of bitter gourd honey to diabetic rats caused substantial reductions in AST, ALT, creatinine, and urea levels [159]. CCl_4_ administration resulted in an increase in lactic acid dehydrogenase, liver enzymes, blood glucose, urea, uric acid, and serum creatinine. It appears that the use of carob honey has been found to have an important effect on protein carbonyl formation, MDA, and advanced protein oxidation products levels and decreases antioxidant enzyme levels. According to one study, these changes were significantly improved by carob honey, both before and after CCl_4_ administration [160] (Table 2).

### 4.8 Neuroprotective effects

The central nervous system (CNS) is impacted by a broad range of neurodegenerative diseases, which result in a disruption of neuronal connectivity and communication that is essential to sensory, motor, and cognitive functions like vision, hearing, movement, speech and language, memory, and others. The continuous deterioration of synapses and axons, which ultimately results in neuronal death, is a hallmark of this breakdown in neural connection. As the world's population ages, it is anticipated that the number of cases of dementia and neurodegeneration would increase sharply, endangering global healthcare infrastructures [161]. Regular use of honey eases anxiety and enhances excitatory effects on the CNS [162] (Figure 5, Table 2).

The neuropharmacological effects of honey were evaluated, and the results showed that honey potentially lowers locomotion and rearing behaviors in novelty-induced behaviors and amphetamine-based locomotor activities [163]. It has been reported that lead exposure decreases exploratory and locomotor behaviors, hinders memory, increases anxiety, enhances lipid peroxidation, and reduces antioxidant activity. However, co-administration of honey in the presence of lead exposure reduced neurotoxicity and led to improvement in memory function, as shown by the decreased latency period and enhanced time spent in the target quadrant in honey-fed animals compared to lead-exposed rats. In addition, honey enhances exploration and locomotion behavior and decreases anxiety in lead-exposed animals [164]. According to one study, kainic acid (KA) has been found to impose additional neuronal degeneration in the piriform cortex and heightens the predilection to seizures in comparison with control animals. However, pre-treatment with Tualang honey reduced the KA-induced neuronal degeneration in the piriform cortex. In the open field test, KA-treated animals showed an increase in locomotor activity and hyperactivity, which was reduced by honey pre-treatment [165].

Rats with Alzheimer's disease and hippocampal damage showed additional noteworthy errors during the Y-maze test compared to the control and other rats. Similarly, neurodegeneration and MDA increased in the Alzheimer's disease group whereas in all defensive and therapeutic groups particularly Iranian thyme honey, they decreased. On the other hand, total antioxidant as well as the number of normal cells increased, and healthy neurons were seen in all parts of the hippocampus as well as cortex [166]. A study based on chrysin demonstrated that chrysin neutralizes early oxidative stress triggered by tert-butyl hydroperoxide in the presence of aluminum chloride. However, this led to late necrotic cell death in neuronal SH-SY5Y cells. Studies in animal models of neurotoxicity induced by chronic exposure to aluminum chloride (100 mg/kg/day) for 3 months have verified the antioxidant and neuroprotective effects of chrysin. Chrysin was found to lower the cognitive impairment induced by aluminum chloride and normalize acetylcholinesterase and butyrylcholinesterase activity in the hippocampus [167].

### 4.9 Anti-diabetic activity

DM is characterized by significantly elevated glucose levels caused by issues with insulin production or insulin resistance; some individuals may experience both factors [168]. According to a report from the centers for disease control and prevention (CDC), around 29.1 million people are affected by diabetes annually, making it the seventh leading cause of death in the United States [168].

It seems that natural honey is consumed as a well-known sweetener, often replacing common table sugars, especially in patients with diabetes. Honey has shown noteworthy potential in the management of diabetes and other complications (Figure 5). The role of honey as a powerful adjunct to metformin or glibenclamide in glucose control was examined, and the results of the study indicated that glibenclamide or metformin combined with honey potentially improved glucose control, which was not achieved with these medications alone [169]. These results are further supported by another study that showed that consuming natural honey along with metformin can significantly improve glycemic control [170].

The anti-diabetic and antioxidant potential of gelam honey, ginger, and their combinations were examined. The combination of ginger and gelam honey significantly reduced the activities of the oxidative stress enzymes SOD and CAT, whereas *GSH* levels were significantly increased [22]. Consumption of foods with a high glycemic index is a substantial risk factor for diabetic patients according to previous research [171]. It has been discovered that some types of honey (such as yellow box and acacia) with a relatively high concentration of monosaccharide (fructose) have a lower glycemic index [172], which could make low glycemic index honey a very valuable alternative sweetener [173].

Honey at different doses was administered to diabetic rats and honey (1.0 or 2.0 g/kg) significantly enhanced high-density lipoprotein cholesterol (HDL-C), whereas it significantly minimized the coronary risk index, reduced triglycerides, hyperglycemia, very low-density lipoprotein cholesterol, and cardiovascular risk index. In addition, high doses such as 3.0 g/kg meaningfully reduced triglycerides as well as very low density lipoprotein (VLDL) cholesterol [174]. Honey treatment led to enhancement in CAT, GSH reductase, and total antioxidant status, and GSH significantly diminished in diabetic animals treated with glibenclamide or metformin. Metformin, glibenclamide, and honey significantly enhanced CAT, GSH reductase, and the total antioxidant status. These outcomes suggest that the combination of honey glibenclamide and metformin might offer extra antioxidant potential to these drugs [175]. Another study revealed that MDA and fasting plasma glucose levels increased in diabetic control rats. The levels of GPx and SOD were significantly enhanced in the diabetic control kidneys. Moreover, the total antioxidant status, body weight, CAT activity, reduced GSH, total GSH, and reduced GSH: oxidized GSH ratios were significantly decreased in diabetic control kidneys. Honey significantly increased the body weight, CAT activity, total antioxidant status, and other antioxidant enzymes in diabetic rats [176] (Table 2).

### 4.10 Cardio-protective activity

CVDs are a significant contributor to global morbidity and mortality, with increasing incidence rates and a decrease in the age of onset over time [177]. This group of diseases comprises conditions such as coronary artery disease, hypertension, heart failure, myocardial infarction, and vascular calcification. The onset of CVD can be influenced by a variety of biochemical, genetic, environmental, and lifestyle factors [178]. The cardioprotective potential of honey has been eminent since early times among common people. Various laboratory studies have demonstrated that honey confers a cardioprotective effect by relieving the oxidative stress created by lipid peroxidation. Honey inhibits lipid peroxidation by activating endogenous antioxidant enzymes [179]. Based on the findings of a study on isolated rat hearts, it has been summarized that supplementation of honey in a normothermic ischemia state protects the heart by possessing antiarrhythmic activity, thus reducing infarction size [180]. In a parallel study, oral gavage of natural honey in animal models of ischemia/reperfusion-induced cardiac arrhythmias reduced the amount and extent of ventricular tachycardia. Honey administration also significantly decreased the sum of ventricular ectopic beats. Furthermore, the use of honey lowers the frequency of reversible ventricular fibrillation [181].

Sundarban honey was studied for its potential to protect the heart in animals with myocardial infarction. The results showed that it increased the levels of a cardiac-specific troponin I molecule as well as several cardiac marker enzymes including creatine kinase-MB, AST, LDH, and ALT. The study also found that rats with myocardial infarction had significant increases in serum levels of triglycerides, total cholesterol, and low density lipoprotein cholesterol (LDL-C), along with a decrease in serum HDL-C levels. However, rats that were pre-treated with honey before ischemia showed nearly normal levels of these biochemical assays, demonstrating a protective effect of honey against ischemia in rats [182]. In the ischemic phase, honey (1%) significantly decreased the number as well as duration of ventricular episodes. Honey (1% as well as 2%) significantly reduced the number of ventricular ectopic beats. Moreover, honey decreased the incidence and duration of reversible ventricular fibrillation. During reperfusion time, in the control group, ventricular tachycardia incidence was 73%, although natural honey (1%) reduced it to 22% [181]. During the ischemia, ventricular tachycardia number as well as duration was decreased after treatment with honey (1%). Moreover, honey (1% as well as 2%) significantly decreased number of ventricular ectopic beats. Additionally, honey treatment caused a reduction in the incidence and duration of reversible ventricular fibrillation [183] (Table 2).

### 4.11 Role in dental health

The oral inflammation, for instance, stomatitis is considered to be triggered or aggravated by various factors including bacterial and viral infections, declined immune functions, allergic reactions, radiotherapy, stress, nutritional deficiencies, cigarettes, diseases and genetic backgrounds [184,185]. The therapeutic potential of honey is very effective against gingivitis and other periodontal diseases (Figure 5, Table 2). The anti-bacterial role of honey was evaluated in parallel with commonly used antibiotics, and the findings revealed that the bacterial counts were significantly reduced in the honey treatment groups, which significantly inhibited the growth of all experimental strains [186]. The therapeutic effects of MH, xylitol chewing gum, and chlorhexidine gluconate mouthwash on the level of dental plaque were compared, and the results suggest that honey has a powerful remedial potential in the management of gingivitis and periodontal ailments [187].

Another study was performed using honey to check its oral anti-bacterial activity, efficiency in decreasing dental plaque, and gingivitis levels. The findings of this study indicated a statistically significant reduction in the mean plaque scores and the fraction of bleeding sites [188]. This study is also supported by other reports, which indicate that honey was able to diminish bleeding and the amount of plaque in patients with gingivitis and plaque [188], and effect of 10% forest honey mouthwash on the dental plaque was reported [189]. A study was undertaken with a purpose to compare the effect of honey, chlorhexidine gluconate mouthwash (0.2%) and combination of xylitol chewing gum and chlorhexidine gluconate mouthwash (0.2%) on the dental plaque levels. The results showed that all three groups were powerful in decreasing the plaque, while post-hoc least significant difference showed that the chlorhexidine + xylitol and honey groups were more powerful than the chlorhexidine group only. The outcomes established an important decrease in plaque present in the honey group as well as chlorhexidine + xylitol group for 2–4 weeks when compared to chlorhexidine [190].

### 4.12 Role in eye health

Some medicinal plants and their natural products have proven to have potential therapeutic roles in the management of diseases related to the eye. Studies have shown that honey might be supportive in the prevention of diseases associated with the eye due to its anti-bacterial potential. In this regard, a previous study was conducted to assess the anti-bacterial effect of honey on the ocular flora of patients with dry eyes due to tear deficiency. This is because honey can be effective in reducing colony-forming units in the eyelids and conjunctiva of dry eye [191]. An important study based on curcumin showed its role in the management of diabetic retinopathy through the regulation of genetic pathways involved in the eye [192], and that ginger extract has protective properties against toxicity caused by ethanol in the eyes of rats [193].

A finding has confirmed that application of honey in the injured corneas showed quicker healing of epithelial and showed a significant role in decreased expression of VEGF, IL-12, as well as TNF-α, in injured corneas [194]. This trial aimed to study the safety and effectiveness of topical honey as eye drops in individuals diagnosed with vernal keratoconjunctivitis. To conduct the study, a total of 60 individuals diagnosed with vernal keratoconjunctivitis were selected. These subjects were divided into two groups: with one group receiving honey eye drops, and the other group receiving a placebo, in addition to topical cromolyn and fluorometholone (1%) eye drops topically. The results of the study showed that subjects who used honey eye drops exhibited a significant reduction in redness, eye pressure, and limbal papillae compared with the placebo control group. After the trial, seven individuals in the placebo group and one subject in the honey group displayed limbal papillae. Overall, the study suggested that topical honey eye drops alongside fluorometholone and cromolyn eye drops may be beneficial in treating vernal keratoconjunctivitis [195].

A pioneering study was performed to evaluate the effects of topically used honey on intact corneas, endotoxin-induced keratitis, and surgically induced corneal abrasions. Results showed that histological analysis demonstrated that no morphological changes or inflammation occurred after honey treatment in the naive intact corneas. Moreover, topical application of honey to injured corneas resulted in quicker epithelial healing as well as reduced VEGF expression, interferon-gamma, TGF-β, IL-12, and TNF-α. Honey treatment has also been reported to decrease inflammation in endotoxin-induced keratitis by decreasing the levels of inflammatory cytokines, angiogenic factors, and chemokines [194]. An important finding was that *in vitro* phase, the relative AUC for cyclodextrin-complexed MH was lower than that for uncomplexed honey. Cyclodextrin-complexed honey showed lower minimum bactericidal concentration and MIC values than uncomplexed honey for both *S. epidermidis* and S. *aureus*, but not for *P. aeruginosa*. It was also found that during the *in vivo* phase of the study, no significant changes were observed in the parameters evaluated in either the treated or control eyes [196] (Table 2, Figure 5).

### 4.13 Renoprotective effects

All chronic kidney diseases lead to renal fibrosis, which is rapidly becoming a serious worldwide health concern. Due to drawbacks including low efficacy or extremely harmful side effects, conventional therapies for renal fibrosis now struggle to meet clinical needs. To address these issues, kidney-focused treatment approaches are therefore required [197]. A study compared the effects of a high-cholesterol diet with and without Tualang honey supplementation. According to this study, there was no significant difference in total cholesterol levels between the two groups. However, the group that received Tualang honey supplementation had lower levels of VLDL, LDL-C, and triglycerides compared to the group that did not receive supplementation. Additionally, this study found that Tualang honey supplementation had a positive effect on renal function, as indicated by an improvement in the renal profile and a lower serum creatinine level [198] (Table 2).

Cisplatin-induced kidney dysfunction was measured by an increase in serum creatinine levels. Moreover, animals that were administered honey showed low kidney malfunction. Honey feeding can significantly reduce the damage to tubular epithelial cells, as well as the expression of cytokines and chemokines, and immune infiltration into the kidney caused by cisplatin treatment. These results suggest that honey can protect the kidneys from cisplatin-induced nephrotoxicity by suppressing NF-kB activation and inflammation [199].

A study was conducted to investigate the effects of honey supplementation on renal function and metabolic acidosis in rats fed with a high-fat diet. The results showed that rats fed with a high-fat diet had higher levels of serum creatinine and anion gap, while their serum total calcium level and ionized fraction were lower than those of rats fed with a regular diet. These findings suggest that the kidneys of control rats fed with a high-fat diet were severely affected, showing signs of inflammation, glomerular atrophy, tubular necrosis, and hemorrhage. However, honey treatment appears to have a protective effect, preventing increases in serum creatinine levels and restoring serum levels of total calcium and ionized calcium to levels similar to those in rats fed with a normal diet [200].

Exposure to carbon tetrachloride (CCl_4_) resulted in a significant increase in lipid peroxidation and advanced protein oxidation products, while there was a marked decrease in GSH, ascorbic acid, GPx, and CAT in the liver and kidney tissues. However, administration of carob honey before and after exposure to CCl_4_ resulted in a significant improvement in these changes, suggesting that carob honey may have a protective effect against the harmful effects of CCl_4_ [160]. A duration of 28 days of honey treatment in rats, alongside melamine, increased antioxidant enzyme levels, improved kidney function, and reduced lipid peroxide levels. The kidney cell morphology of the melamine-treated animals was better because of the honey treatment [201].

### 4.14 Anti-obesity effect

Throughout the world, obesity has become more common. Numerous epidemiological studies have shown that obesity is a major risk factor for the development of liver illnesses, CVDs, cancer, type 2 diabetes, and other conditions, which places a significant annual burden on the general public and healthcare systems [202]. To examine whether sucrose, honey, as well as different sugars present in honey, have any role in weight increase, rats were given powdered food as either free of sugar or containing 8% sucrose, a combination of different sugars (8%) same as in honey, or honey (10%). The animals that were fed honey experienced a significant reduction in the whole percentage weight increase compared to those treated with sucrose or mixed sugars, even though their food consumption was similar. Interestingly, the weight gain of rats given honey was comparable to that of rats fed a sugar-free diet, but the rats given honey consumed more food [203]. A study found that rats fed with honey gained less body weight and had lower epididymal fat weight compared to rats not given honey. The study also reported lower levels of serum leptin and triglycerides, and higher levels of non-HDL cholesterol in honey-treated animals. Based on the outcomes of the study you mentioned earlier, it appears that honey may be more effective than sucrose in reducing weight gain and adiposity. This effect may be due to the lower food consumption observed in the honey-fed rats. Additionally, honey may promote lower serum triglycerides [204].

A recent study investigated the effect of natural honey on various health markers in overweight individuals. The study evaluated the effects of honey on fasting blood glucose (FBG), LDL-cholesterol, total cholesterol, triacylglycerol, HDL-C, C-reactive protein (CRP), and body weight. Honey may have positive effects on body fat, weight, and cholesterol levels. It is important to note that it can help minimize total cholesterol, triacylglycerol, and FBG levels, while also increasing HDL-C levels in individuals with normal values. For those with raised variables, it is encouraging to see that honey can help decrease LDL-C, total cholesterol, CRP, and triacylglycerol levels [205].

Researchers have investigated the potential role of honey in body weight and blood biochemical parameters in diabetic subjects. A total of 48 type II diabetes patients were randomly allocated into two groups: the honey group and the control group without honey. These findings are quite promising, and it was observed that the use of honey for 8 weeks could lead to meaningful decreases in body weight, total cholesterol, low-density lipoprotein cholesterol, and triglyceride levels, while also increasing HDL-C levels. These results suggest that honey could be a useful addition to the management of blood lipids in patients with diabetes [206] (Table 2).

### 4.15 Anti-hypertensive effect

Hypertension is the foremost cause of the worldwide disease burden, causing 10.4 million deaths in 2017 [207], and is responsible for 22.3% of the population’s attributable load of CVD [208]. Honey has been found to have antihypertensive effects, which can help inhibit pathogenesis (Table 2). Results demonstrated that Control spontaneously hypertensive rats (SHRs) had significantly higher renal MDA levels and systolic blood pressure. The mRNA levels of nuclear factor erythroid 2-related factor 2 (Nrf2) and GST were significantly downregulated, whereas the activities of GST, total antioxidant status, and CAT levels were enhanced in the kidneys of the control SHR. Honey treatment reduces systolic blood pressure and MDA levels in SHR [209].

A study was conducted with 4,561 participants, and blood pressure was measured at least twice. To investigate the potential link between honey consumption and prehypertension, researchers employed multiple logistic regression models. The study found some interesting results regarding the relationship between honey intake and prehypertension. After adjusting for confounding factors, the odds ratios and 95% confidence intervals for prehypertension varied across different frequencies of honey intake were 1.00 (reference) for nearly never, 0.76 (0.62, 0.92) for ≤6 times a week, and 0.84 (0.63, 1.12) for more than 7 times per week in women, and 1.00 (reference) for rarely, 1.17 (0.96, 1.41) for less than 6 times a week, and 1.25 (0.86, 1.84) for more than 7 times a week in men. A study found a correlation between moderate honey intake and a lower prevalence of prehypertension in women, but not in men [210].

### 4.16 Anti-allergic effect

The most prevalent chronic inflammatory skin condition is atopic dermatitis (AD). Approximately 80% of disease cases usually begin in childhood or infancy, with the remaining instances emerging in adulthood. Individual trajectories of the disease are unpredictable, and its natural course exhibits a great degree of variation. Dry, sensitive skin, and isolated or widespread eczematous lesions, typically accompanied by intense itching, are the hallmarks of AD [211]. The role of honey in AD treatment was also investigated. Honey can potentially help with AD. There was a noticeable improvement in lesions after treatment with MH. It was also reassuring that there were no significant variations in the skin staphylococci after the treatment. According to clinical findings, MH decreases IL-4-based CCL26 formation in HaCaT cells. This potential partially disappeared; however, it persisted substantially when hexane and methanolic extracts of honey were used. Mast cell degranulation may be repressed following honey treatment, suggesting that it could be a useful treatment option based on different mechanisms [212].

To scrutinize the possible role of honey, an olive oil and beeswax mixture was used in subjects with psoriasis vulgaris (PV) or AD. In the honey mixture group (honey, beeswax, and olive oil (1:1:1), patients with dermatitis displayed significant improvement after 2 weeks. In psoriasis, five out of eight patients presented a substantial response to honey. In subjects using clobetasol propionate, 5 out of 10 patients exhibited no deterioration upon a 75% decrease in corticosteroid with the use of mixture. Overall, finding an advocate that a honey mixture seems valuable in the management of PV and dermatitis [213], and other studies advocate that honey could be a helpful natural remedy for those suffering from allergic rhinitis [214].

A study was designed to explore the role of anti-bacterial medical media honey in patients who continue to suffer from allergic fungal rhinosinusitis resistant to conventional medical treatment. As a group, the 34 patients who completed the study displayed insignificant improvement in the treated nostrils vs control nostrils. However, nine patients who responded to honey treatment relative to their control side responded very well. The MH did not change the culture outcomes in the ethmoid cavities after 30 days of treatment; however, patients who completed the Sino-Nasal Outcome Test-22 questionnaire designated global improvement in their symptoms while taking the honey spray [215].

### 4.17 Effect on the respiratory system

Worldwide, pulmonary diseases such as acute respiratory infections, chronic obstructive pulmonary disease, bronchial asthma, and lung cancer are associated with significant rates of illness, death, disability, and expensive treatment costs [216]. This study investigated the effects of aerosolized honey on airway tissues, and the results showed that induction with ovalbumin caused changes in the submucosal regions of the airway, epithelium, and mucosa. Aerosolized honey treatment has the potential to inhibit goblet cell hyperplasia. This may be a promising approach for the treatment of respiratory conditions. Interestingly, research suggests that aerosolized honey could be an effective treatment for asthma in both rabbits and humans. This study found that it was able to efficiently treat and control asthma in rabbits, and there is hope that it could have similar benefits for humans [92].

The effect of gelam honey on the lung histopathological architecture of allergic asthma mouse models was investigated. The mast cell number, epithelium thickness, and mucus expression were improved compared to the group with the exception of subepithelial smooth muscle thickness. In addition, gelam honey substantially reduced the infiltration of inflammatory cells and beta-hexosaminidase levels in bronchoalveolar lavage fluid. A study based on these findings concluded that a high dose of gelam honey improved the histopathological tissue architecture of allergic asthma animal models [217]. The modulatory effects of royal jelly, honey, and propolis have been previously reported [218]. After 4 weeks of exposure to paraquat, the number of tyrosine-hydroxylase immunopositive neurons and GPx activity in the midbrain were reduced in animals. The lungs from group paraquat presented suggestively reduced activity of GST and SOD. Consumption of Tualang honey improved the toxic effects observed in the lungs and midbrain. These results suggest that Tualang honey may protect against paraquat-induced toxicity in the rat lung and midbrain [219].

### 4.18 Gastroprotective effect

Gastritis and gastric ulcers are common diseases, and various factors are involved in gastric ulcers, including inappropriate use of aspirin, *Helicobacter pylori* infection, cigarette smoking, excessive drinking, or even stress [220–222]. Natural honey and Nigella sativa seeds are equally efficient in healing gastric ulcers, such as cimetidine [223], and the gastric cytoprotective properties of natural honey have been well reported [224]. MH significantly decreased the ulcer index, conserved gastric mucosal glycoproteins, and fully protected the mucosa from lesions. This promising discovery could potentially lead to the development of new treatments for ulcers [225].

Oral gavage of honey or sucralfate before ammonium hydroxide treatment minimized the severity of gastric mucosal lesions and changes in nonprotein sulfhydryl levels. Furthermore, a combination of sucralfate or a low dose of honey (0.312 g/kg) pre-treatment afforded enhanced protection (58 and 77%) than that achieved with either of them given alone [226]. It is interesting to note that oral pre-treatment with Alimento Supervis, Alimento Mieleucalipto, and honey (2 g/kg) once daily for 7 successive days inhibited indomethacin-induced gastric lesions in rats. This effect is achieved by decreasing the gastric microvascular permeability, ulcer index, and myeloperoxidase activity [227]. The healing effect of wild honey and its combination with turmeric has been confirmed in gastric ulcers [228].

### 4.19 Effect on skin health

The epidermis, dermis, and subcutaneous layers make up the skin, the biggest organ in the human body. It guards against physical harm, the external microbiological environment, ultraviolet (UV) radiation, and moisture loss. Numerous factors, such as personal characteristics, lifestyle decisions (such as drinking alcohol and not getting enough sleep), environmental factors (such as UV rays and pollutants), and different illnesses, are linked to issues with skin health [229]. It was reported that 8 out of 10 patients with dermatitis showed significant improvement after 2 weeks of using the honey mixture, and a total of 5/11 patients pre-treated with betamethasone esters exhibited no deterioration upon 75% decrease in corticosteroid by the use of mixture. Honey mixture provides a beneficial impression for the management of dermatitis [213].

It is interesting to know that the role of topical use of crude honey in the treatment of seborrheic dermatitis and dandruff was investigated. One group of patients used topical honey once weekly, and the other group was used as the control. All subjects responded appropriately to the application of honey. Scaling and itching were comforted within a week, and the skin lesions were treated completely within 2 weeks [230]. The efficacy of Honevo, a kanuka honey (90% medical grade), and 10% glycerin as a treatment approach for facial acne was investigated. A higher percentage of participants in the honey product group (7.6%) showed significant improvement in their Investigator's Global Assessment score at week 12 compared with the control group (1.9%). This randomized controlled trial did not indicate that adding kanuka honey and 10% glycerin to anti-microbial soap treatment is more effective than the use of anti-bacterial soap only in the treatment of acne [231]. Manuka and clover honey have shown anti-viral activity against varicella-zoster virus. These results suggest that honey has an important role *in vitro* anti-varicella zoster virus (anti-VZV) activities. Honey can be an excellent remedy for treating zoster rashes. It is easily available, inexpensive, and suitable for skin applications [232] (Table 2).

### 4.20 Effect on the reproductive system

Human health is greatly impacted by reproductive diseases, particularly the health of women. Moreover, the rate of infertility is also increasing. Relatively few new reproductive illness diagnostics or treatments have been developed in recent decades, nevertheless. The development of comorbidity is increased by the difficulty in diagnosing and treating reproductive disorders. Furthermore, the acute and chronic healthcare burden brought on by reproductive diseases is expected to keep rising due to a historical dearth of study on reproductive health and disease [233]. A previous study concluded that Malaysian honey, 1.2 g/kg daily significantly enhanced the count of epididymal sperm without affecting reproductive hormones and spermatid count. These results suggest that oral administration of honey at this dose for 4 weeks may increase spermiogenesis in adult rats [234].

A study explored the protective role of Persian honey during post-ischemia reperfusion in testis injury. According to this study, the serum levels of follicle-stimulating hormone and luteinising hormone were significantly higher in the carbohydrate + ischemia-reperfusion and honey + ischemia-reperfusion groups. In addition, serum testosterone levels are higher in the vitamin C + ischemia-reperfusion and honey + ischemia-reperfusion groups. This study indicated that honey reduces cellular damage as well as apoptosis, with substantial defensive potential for reproductive hormone synthesis [235].

Honey treatment in prenatal restraint stress enhanced epididymis and testis weights, as well as enhanced the percentage of abnormal spermatozoa [236]. Compared to the control group, supplementation with honey significantly improved the viability index and post-thaw sperm motility and showed a positive role in membrane integrity as well as intact acrosome percentage. For all semen parameters, the higher concentration (5%) and lower concentration of honey (1%) did not display substantial differences compared with the control [237]. Honey caused beneficial effects in lowering the rise in cortisol as well as in boosting the decrease in progesterone levels induced by diverse intensities of jumping exercise in female rats [238]. The role of pine honey in several concentrations of solutions on cryopreservation as well as fertilization capability of spermatozoa of common carp was investigated. The extenders comprising 300 mg/mL pine honey group exhibited both maximum post-thaw motility of 75.3 ± 5.1%, hatching ratio of 42.6 ± 4.2%, and motility duration of 47.3 ± 2.5% than other cryopreserved groups [239].

### 4.21 Radioprotective effect

The radioprotective potential of Kelulut honey from stingless bees (*Trigona* sp.) on zebrafish embryos was investigated. Kelulut honey has been shown to decrease coagulation potential. The embryos developed a combination of abnormalities, viz., body curvature, microphthalmia, microcephaly, and pericardial edema. At 96 h post-irradiation, Kelulut honey decreased the body curvature potential in irradiated embryos. Kelulut honey has been shown to protect against organ-specific abnormality [240]. Protection of honey against X-ray-based genotoxic damage was also investigated. Blood lymphocytes from healthy volunteers were categorized into two groups. The lymphocytes were treated with 10% diluted honey. Both the study and control groups were treated with high-dose X-ray at the 48th hour of culture. Micronucleus frequency was significantly reduced in the study group compared to that in the control group. Chromosomal breakage was significantly reduced in the study group compared to the control group. This study demonstrated that genotoxic damage in healthy group blood lymphocytes induced by X-ray irradiation may be inhibited or alleviated by Anzer honey *in vitro* [241]. The radioprotective potential of propolis supplementation was examined in breast cancer patients undergoing radiotherapy. The results showed that in patients supplemented with propolis + radiotherapy, propolis showed the capability to decrease radiation-induced DNA damage. Moreover, propolis supplementation during radiotherapy showed a noteworthy downregulation of RRM2 levels. Propolis supplementation with radiotherapy provides measurable protection against DNA damage [242].

## 5 Synergistic effects of honey in combination with other compounds in disease prevention

The anti-bacterial potential of the powder of *Thymus Ciliatus* as well as wild carrot honey when used as a combination for the measurement of MIC against three pathogenic bacteria revealed that when honey as well as thyme powder are used as a combination, a decrease in the MIC values occurs, which might be due to its synergistic effect [243]. MH and oxacillin act synergistically to prevent methicillin-resistant *Staphylococcus aureus* (MRSA) infection. Moreover, MH upturned oxacillin resistance in MRSA, and downregulation of mecR1 was observed in cells exposed to MH [244].

Synergism between Medihoney and rifampicin against MRSA and *Staphylococcus aureus (S. aureus)* clinical isolates was observed. Additionally, the Medihoney/rifampicin combination prevented the presence of rifampicin-resistant *S. aureus in vitro* [245]. MH showed a synergistic action with vancomycin against *S. aureus* biofilms and an additive interaction with gentamicin against *P. aeruginosa* biofilms [246]. Honey-ginger powder extract combinations showed the maximum mean inhibition compared to the use of ginger extracts or honey individually [247]. When tested separately, the MIC of the four types of honey ranged from 12 to 18% and that of royal jelly was 4%. When combined with royal jelly, each honey type examined displayed a higher than 90% drop in MIC using 3% (v/v) royal jelly, a 66.6% decrease in MIC using 2% (v/v) royal jelly, and a 50% MIC drop using 1% (v/v) royal jelly [248]. The average MICs of carvacrol, cinnamaldehyde, and honey were 0.01–0.6, 0.82–0.01, and 62.5–250 μg/mL, respectively. A synergistic effect was observed between some drugs against 70% of the isolates [249]. When honeydew honey (100%) with Vit C (100 mg/g of honey) was used in the treatment in a wound biofilm model, comprehensive abolition of nearly all bacterial isolates was observed [250]. The results indicated substantial anti-bacterial activity of ginger and honey [251]. Ginger starch, which was the focus of the study, displayed an amazing additive effect on the anti-bacterial potential of honey against the bacteria used [252]. The inhibition zone of a mixture of honey and garlic was significantly higher than that of garlic or honey alone [253]. Mixture of honey and *Allium sativum* showed synergistic anti-microbial potential and higher wound-healing activity [254]. Curcumin, whey, and MH have powerful effects on bacterial strains. It is especially impressive that MH and curcumin could completely inhibit the growth of all the tested bacterial strains. At these levels, the combination of honey and whey protein isolate was more effective against certain bacterial strains than each substance alone. This combination has improved and/or synergistic anti-microbial effect [255].

**Figure 6:**
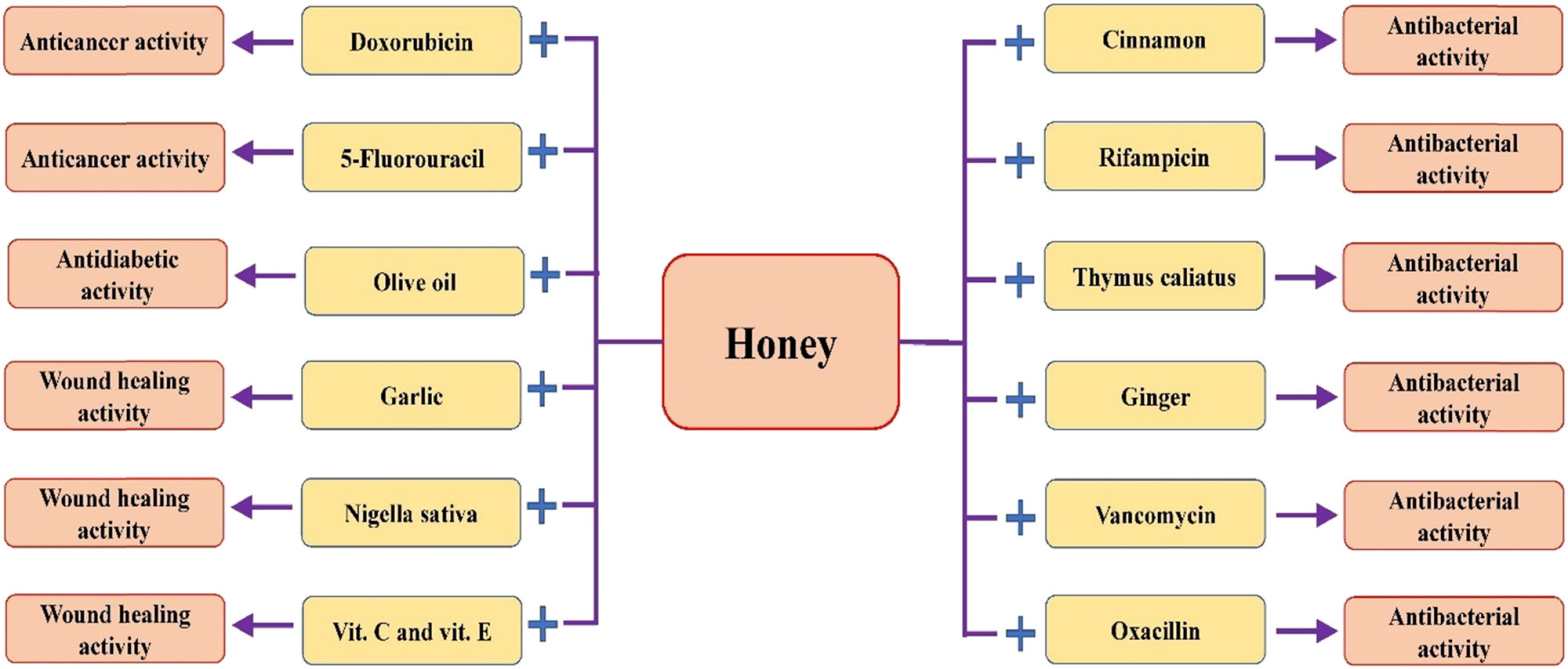
Synergistic effects of honey in combination with other compounds.

The mixture of honey and *Nigella sativa* (*N. sativa*) showed better wound healing potential [256] (Table 3). The MIC for the three types of honey without *N. sativa* against *P. aeruginosa* ranged between 46 and 50%. The addition of *N. sativa* resulted in synergistic bactericidal activity [257] (Figure 6). Thyme honey and olive oil, especially when used together, have potential benefits for patients with diabetes. They can improve blood glucose levels and protect against metabolic changes and complications associated with diabetes [258].

**Table 3:** Synergistic effects of honey in combination with other compounds in disease prevention

Honey types	Natural compounds/drugs	Activity	Outcome of the study	Refs.
Carrot honey	*Thymus Ciliatus*	Anti-bacterial	When honey as well as thyme powder are used as a combination, a decrease in the MIC was observed	[243]
MH	Oxacillin	Anti-bacterial	MH as well as oxacillin acted synergistically to prevent MRSA	[244]
Medihoney	Rifampicin	Anti-bacterial	Honey/rifampicin combination prohibited the presence of rifampicin-resistant *S. aureus*	[245]
MH	Vancomycin	Anti-bacterial	MH showed a synergistic action with vancomycin against biofilms	[246]
Ethiopian honey	Ginger	Anti-bacterial	Honey-ginger powder extract combinations showed the maximum mean inhibition	[247]
Algerian honey	Royal jelly	Anti-bacterial	When combined with royal jelly, honey displayed higher than 90% drop in MIC	[248]
Iranian honey	Carvacrol and cinnamaldehyde	Anti-bacterial	A synergistic effect was seen between drugs against isolates	[249]
Slovakia honey	Vit C	Anti-bacterial	A mixture of honey as well as Vit C was partly effective against *Enterococcus faecalis* (*E. faecalis*), while only honey presented no anti-bacterial potential	[250]
Indian Dabur honey	Ginger	Anti-bacterial	Combined extracts were most powerful against *S. aureus*	[251]
Algerian honey	Ginger	Anti-bacterial	Additive effect on the anti-bacterial potential	[252]
Tazma honey	Garlic	Anti-bacterial	The inhibition zone of a mixture of tazma honey and garlic was meaningfully higher than garlic or tazma honey alone	[253]
Euphorbia honey	Garlic	Wound healing	The mixture displayed higher wound-healing activity	[254]
MH	Curcumin and whey protein isolate	Anti-bacterial	MH and curcumin showed 100% inhibition against tested bacteria. A mixture of honey and whey protein isolate was more powerful against *Shigella sonnei*	[255]
Iranian honey	*Nigella sativa*	Wound healing	A substantial decrease in wound surface area was noticed	[256]
Algerian honey	*Nigella sativa*	Anti-bacterial	Addition of *N. sativa* caused synergistic bactericidal activity	[257]
Thyme honey	Olive oil	Anti-diabetic	Improves blood glucose levels and defends against metabolic changes	[258]
Iranian honey	Cinnamon	Anti-bacterial	The mixture of honey and cinnamon showed anti-bacterial effects	[259]
Medical grade honey	Vitamins C and E	Anti-fungal	Combination showed anti-fungal effects	[260]
MH	5-Fluorouracil	Anti-cancer	MH synergistically increased the chemotherapeutic effects of 5-fluorouracil	[261]
MH	Doxorubicin	Anti-cancer	MH or combined treatment-encouraged apoptosis by meaningfully downregulated expression of anti-apoptotic protein and upregulated expression of pro-apoptotic protein	[262]

An effective synergistic effect of honey and cinnamon was observed against *Streptococcus mutans (S. mutans)* [259]. Moreover, vitamin supplements or other components of Mesitran may improve the anti-fungal activity of honey [260]. Compared to 5-fluorouracil alone, honey synergistically increased the chemotherapeutic effects of 5-fluorouracil, by promoting apoptosis [261] (Figure 6, Table 3). MH and doxorubicin have synergistic effects on apoptosis. Moreover, MH or combined treatment-encouraged apoptosis was confirmed by a meaningfully decreased expression of anti-apoptotic protein and increased expression of pro-apoptotic protein [262] (Table 3, Figure 6).

## 6 Honey in modern medicines

Nowadays, various infectious diseases are commonly spreading around the world due to environmental pollution and related consequences. These diseases are not well controlled by the present drug treatment. Antibiotics are failing because of bacterial resistance. However, people believe that herbal medicines are more effective and safer. Therefore, traditional herbal remedies have been recommended for treatment purposes. Herbal medicines are often used in combination, fused with honey, or alone for curing different types of diseases. Today, modern formulations of these medicines exist in the form of capsules, tablets, powders, and granules [263].

In addition to its importance in traditional medicine, natural honey has gained recognition in modern medicine over the last few decades as a result of numerous research groups’ laboratory and clinical studies on the subject. Various bacterial species, along with a number of fungal and viral species, are said to be inhibited in their growth by honey. Honey has a strong antioxidant capacity that is advantageous in various disease states because of a number of constituents, such as phenolics, peptides, organic acids, enzymes, and products of the Maillard reaction. Honey has also been used to treat a number of neoplastic, inflammatory, cardiovascular, and gastrointestinal disorders. Eye disorders, throat infections, bronchial asthma, TB, hiccups, thirst, dizziness, exhaustion, hepatitis, worm infestation, constipation, piles, eczema, wound healing, and ulcers are just a few of the ailments that can be treated with fresh bee honey [264]. Nowadays, honey-based gel, dressing, and ointments are used but only limited clinical trial data or other levels of suggestion for the claims made for these specific commercial formulations are publicly available, the efficiency of such gels can be derived from various studies [265–271]. Moreover, the evidence of the efficiency of honey-based dressings has been reported [272–274] and MH -based creams is evidently obvious from studies [93,275].

## 7 Beekeeping and honey production

Beekeeping serves not only as a source of honey but also plays a crucial role in ensuring food and nutrition security, as it is responsible for pollinating approximately 30% of the world’s food production [276]. This makes beekeeping essential for the sustainability of global food resources. Additionally, agricultural management practices are being adopted within beekeeping as strategies to adapt to climate change [277–279]. Beekeeping is a profitable enterprise that greatly boosts and diversifies the incomes of several rural households. For example, honeybee pollination has been shown to improve the quality and yields of numerous major crops, including tomatoes, watermelon, and citrus sinensis [280]. The honeybees rely on natural feed or additional nutrition to generate honey. The nectar of plants, plant secretions, or the waste products of plant-sucking insects are among the natural food sources for honeybees. Honey is categorized into two types based on the natural food sources of honeybees: nectar or blossom honey, and honeydew honey. Nectar honey is made by honeybees that collect nectar from plants, and it is abundant in pollen. On the other hand, honeydew honey is produced when honeybees utilize the secretions from living plant parts [281]. The main components of honey are sugars and water, while the minor components consist of acids, proteins, amino acids, enzymes, flavonoids, phenolic acids, minerals, and vitamins [282–284]. Honeybees can either connect their tongue to the wall of lengthy corollas and drink the nectar along the sides of their tongues, or they can dip their hairy tongues or capillary loading when lapping it. The feeding mechanism of honeybees is revealed to be a multipurpose instrument that may alternate between sucking and lap nectar according to the instantaneous ingesting efficiency, which is dictated by the interaction of sugar content and nectar–mouth distance. These adaptable feeding strategies give honeybees exceptional flexibility in a variety of foraging habitats and enable them to efficiently harvest nectar from a greater variety of floral resources than previously thought [285].

## 8 Conclusion and future prospective

It is widely recognized that medicinal plants and their components can promote health by regulating physiological and biochemical processes. The health-promoting properties of natural honey have been discussed in various religious texts and traditional medicine. Medicinal plants and their components are well-known for their health-promoting activities that regulate physiological and biochemical processes. The health-promoting benefits of natural honey have been discussed in various religious books and traditional medicine. Honey-based treatment practices are widely used in Ayurveda and Chinese medicine, and are considered safe, effective, and broad-range remedies in health management. Its health benefits are attributed to its numerous valuable ingredients. The antioxidant, anti-inflammatory, and anti-microbial potential of honey is well documented, and its role is attributed to the enhancement of antioxidant enzyme activities and the reduction in oxidative stress levels. Experimental studies have also confirmed that honey plays a significant role in tumor inhibition through the suppression of angiogenesis, cell cycle, and induction of apoptotic pathways. However, more research is required to understand the specific role of honey in the regulation of genetic pathways and its mechanism of action in disease treatment and prevention. Furthermore, research should also be conducted to recognize each specific component present in honey that plays a significant role in the cure of diseases, including cancer.
